# Application of a molecular networking approach using LC-HRMS combined with the MetWork webserver for clinical and forensic toxicology

**DOI:** 10.1016/j.heliyon.2024.e36735

**Published:** 2024-08-31

**Authors:** Romain Magny, Yann Beauxis, Gregory Genta-Jouve, Emmanuel Bourgogne

**Affiliations:** a Laboratoire de Toxicologie, Fédération de Toxicologie, AP-HP, Hôpital Lariboisière, 75006, Paris, France; b Université Paris Cité, CNRS, CiTCoM, 75006, Paris, France; c Université Paris Cité, Faculté de santé, Laboratoire de toxicologie, 75006, Paris, France; d USR 3456 CNRS LEEISA, Guyane, France; e Laboratoire de Pharmacologie, AP-HP, Hôpital Bichat, 75018, Paris, France

**Keywords:** Molecular network, Drug identification, Clinical toxicology, Untargeted screening, High-resolution mass spectrometry, *In silico* data analysis

## Abstract

**Backgrounds and aims:**

In toxicology, LC-HRMS for untargeted screening yields a great deal of high quality spectral data. However, there we lack tools to visualize/organize the MS data. We applied molecular networking (MN) to untargeted screening interpretation. Our aims were to compare theoretical MS libraries obtained *in silico* with our experimental dataset in patients to broaden its application, and to use the MetWork web application for metabolite identification.

**Methods:**

Samples were analyzed using an LC-HRMS system. For MN, data was generated using MZmine, and analyzed and visualized using MetGem. MetWork annotations were ﬁltered and this ﬁle was used for annotation of the previously obtained MN.

**Results:**

155 compounds including drugs found in patients were recorded. Using this dataset, we confirmed in 60 patients intake of tramadol, amitriptyline bromazepam, and cocaine. The results obtained by the reference methods were confirmed by MN approaches. Eighty percent of the compounds were common to both conventional and MN approaches. Using MetWork, metabolites and parent drugs such as amitriptyline, its metabolite nortriptyline and amitriptyline glucuronide phase 2 metabolites were anticipated and proposed as putative annotations.

**Conclusion:**

The workflow increases confidence in toxicological screening by highlighting putative structures in biological matrices in combination with CFM-ID (Competitive Fragmentation Modeling for Metabolite Identification) and MetWork to extend the annotation of potential drugs even without a reference standard.

## Highlights

• In toxicology, analysts are confronted with complex problems, which have clinical consequences.
• Untargeted LC-HRMS screening remains a challenge.
• Molecular networking (MN) for screening interpretation was applied in real patients for drug annotation.
• When combined with CFM-ID and the MetWork webserver to extend a drug's annotation, MN could help clinicians to explain toxicity.

## Graphical abstract

**Figure:**
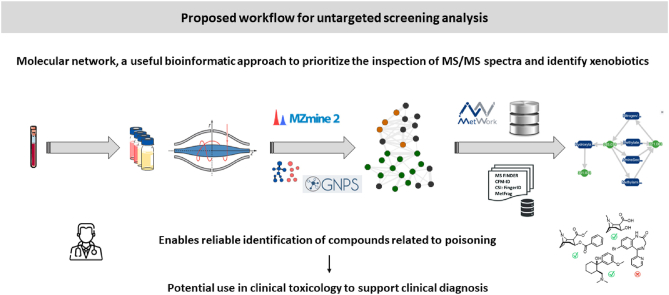

## 1 Introduction

In therapeutic drug monitoring and toxicology, the bioanalyst frequently faces difficult issues that have potentially important clinical consequences. In drug testing, screening is used to confirm the presence of drugs of abuse or therapeutic drugs in bodily specimens such as blood, plasma, and urine. Today, in clinical and forensic toxicology, the first step is to identify the compounds to be quantified, as they are often unknown in advance. This plays a major important role in forensic and clinical toxicology (drug dependence, pain management, adherence testing). Conversely, for rapid therapeutic drug monitoring, known drugs are identified and quantified to assess treatment efficacy. Typically, these drugs have narrow therapeutic ranges. Unpredictable levels of the drugs in the patient's blood may further complicate the issue. Many variables contribute to the unpredictable pharmacokinetics of a drug, including drug-drug interactions, age, race, and gender [1,2].

Risk factors for hospital admission increase with age or polypharmacy. Depending on the study, drug-drug interactions are estimated to account for 2–5% of hospitalizations in elderly patients and 1 % of hospitalizations in the general population [3,4]. The number of prescribers involved also has a significant impact on the risk of drug interactions. Among individuals with five or more prescribers, approximately one-third of polypharmacy patients are exposed to potential drug-drug interactions [5]. Adverse drug reactions are a major source of morbidity in about 10 % of outpatients and in 10–20 % of inpatients. They account for 5 % of all hospitalizations. Drug-drug interactions are also a significant cause of death among hospitalized patients [6].

Currently, to fulfill these drug quantification and identification tasks, instruments range from automated workstations to front-end mass spectrometry (MS) instruments. Immunoassay kits may potentially not work with samples that are not specified by the manufacturer, and a conventional immunoassay will only quantify one target analyte. Immunoassay results are also considered presumptive until confirmed by higher resolution techniques like gas or liquid chromatography-MS (GC-MS, LC-MS) methods. As an example, for opiates, conventional immunoassays commonly used for urine drug screening are sensitive to only a small number of natural opioids or to only a single drug, and show variable degree of cross-reactivity with their phase 2 metabolites. The detection limits of these immunoassays may be insufficient for medical practice, thus hampering clinical interpretation of immunoassay results. The implementation of hyphenated MS techniques in the clinical laboratory has been driven by these limitations of immunoassays [7]. For nearly two decades, assays based on LC-MS/MS have been adapted by many clinical laboratories, and GC– or LC–MS are now the gold standard for pharmacological/toxicological analysis as they provide high specificity, sensitivity, and universality. LC-MS is currently the reference method for quantification or identification of drugs in biological samples [[8], [9], [10]], with recent applications focusing more on “personalized” or “precision” medicine [11]. For quantitative targeted analysis of small molecules, LC-QqQ low-resolution MS has been identified as the technique of choice [[11], [12], [13]]. Nevertheless, due to the diversity of pharmacologically or toxicologically relevant compounds, the uncertainty of target substances and the specificity of samples, untargeted screening approaches have long been favored by analytical chemists. In addition, it can be relatively easy to comment on a targeted assay result, but the main problem for doctors remains the day-to-day management for poly-prescribed patients. To answer their questions on potentially harmful drug-drug interactions, it is necessary to know both the pharmacokinetic profiles of the drugs and the numerous associated treatments. Therefore, the targeted analysis is only the tip of the iceberg and, obviously, the questioning of doctors is quite different in the search for exhaustive screening strategies to identify drugs.

Screening strategies using low-resolution mass spectrometers can be categorized as targeted or comprehensive non-targeted screening methodologies. Targeted approaches are used to screen for a defined list of analytes by means of Selected Ion Monitoring (SIM) or Selected Reaction Monitoring (SRM), while analytes not selected/included *a priori* will not be detected. Untargeted approaches comprise Systematic Toxicological Analysis (STA) or General Unknown Screening (GUS) and use full scan acquisition, data-dependent or data-independent MS/MS acquisition and reference libraries for identification of a large range of drugs, poisons, and/or their metabolites. For (multi)target screening, so-called multi-analyte methods are usually employed. These represent a balance between the use of as few methods as necessary for as many analytes as possible [14]. By providing accurate mass measurements with sub-ppm errors, High-Resolution MS (HRMS) instruments not only improve the detection of metabolites by discriminating between isobaric compounds, but also help in their identiﬁcation or structural characterization. LC-HRMS analyzers have become more and more advantageous for screening and identifying chemical compounds. These Time-of-Flight (ToF) and Orbitrap high-resolution instruments are currently the reference method for screening, allowing untargeted analysis and are also being increasingly used in quantitative bioanalysis [15]. The applications of LC-HRMS are already used with success in screening and identification of prescription drugs, drugs of abuse, pesticides or stimulants [16,17], and also for metabolomic [18], lipidomic [19], proteomic studies [20]. Metabolomic applications cover numerous fields, including toxicology [21] and personalized medicine [22].

Untargeted screening may also be a challenge because of the large number of molecules to be detected and the absence of available standards. LC-HRMS generates a large volume of high-quality spectral data, but we lack tools to visualize and/or organize the MS data of these compounds. Molecular networking (MN) is used to organize and display graphically untargeted tandem MS (MS/MS) data [23]. A molecular network is a visual representation of structural similarity between MS profiles acquired during an untargeted MS/MS analysis. Each node represents an ion and the corresponding fragmentation spectrum, and the connections between nodes indicate significant similarities between spectra. The MN approach provides valuable knowledge about drug metabolism by propagating structural information through the network. MN has already been used in various fields such as toxicology and is successfully implemented to support clinical and forensic investigations [[24], [25], [26]].

Here, we applied MN to untargeted screening interpretation with a view to helping clinicians better understand drug-drug interactions and to explain the absence of efficacy or potential toxicity in the treatment of patients. To achieve these objectives, several steps were taken: (i) build a mass spectral library of drugs found in cases of poisoning and used by patients admitted to the clinical wards, both in collision-induced dissociation (CID) and high collision dissociation (HCD), and to use this database for drug identification in hospitalized patients; (ii) compare theoretical mass spectral libraries obtained by *in silico* fragmentation with the current database to broaden its application; (iii) use MetWork to annotate putative structures in the network.

## 2 Materials and methods

### 2.1 Chemicals and reagents

Methanol, acetonitrile, isopropanol, water of LC-MS grade (J.T. Baker, Phillipsburg, NJ, USA) were used for protein precipitation sample preparation and LC mobile phase preparation. Commercial drug standards and/or certified reference materials were hospital stock solutions or from Ceriliant (Sigma-Aldrich, Lyon, France). They are listed in Table S1 in the Supplementary Materials.

### 2.2 Sample preparation

For the database, each standard drug was diluted in methanol to 1 mg/mL and further diluted to 100 μg/mL in methanol. 5 μL was finally injected onto the LC-HRMS system. Plasma was taken from the bottom of tubes intended for analyses carried out as part of patient management. No mandatory formal requirements were therefore required. Nevertheless, where possible, patients were given information and their consent was obtained [27]. Blood samples were collected in heparinized tubes, and plasma was separated by centrifugation at +4 °C within 1.5 h of arrival at the hospital laboratory. Plasma was stored at −20 °C until analysis, as recommended. Once centrifuged, 100 μL of plasma was mixed with 100 μL of acetonitrile for protein precipitation. The mixture was sonicated for 30 s, then centrifuged for 10 min (10000 rpm–13000 g). Finally, the supernatant was collected and 5 μL was injected onto the LC-HRMS system.

### 2.3 Data-dependent LC-HRMS/MS analysis

LC-positive electrospray ionization MS analysis was performed on a UHPLC system (ThermoFisher®, San Jose, USA) combined with a LTQ XL Orbitrap® high-resolution MS (ThermoFisher®, San Jose, USA). Chromatographic separation was achieved on an Acquity® (Manchester, UK) xbridge BeH C18 column (150 × 2.1 mm; 1.7 μm). Drugs were eluted using a binary gradient system consisting of 0.1 % formic acid in water as solvent A and 0.1 % formic acid in acetonitrile as solvent B. The eluent was isocratic during 3 min then increased from 5 % B to 100 % B in 7 min, was held at 100 % B for 2 min before returning to 5 % B. The flow rate was kept at 0.2 mL min^−1^. The injection volume was 5 μL and the column oven was set at 40 °C. For the purpose of the database, total run time was 17 min and was extended to 45 min for patient analysis. Database LC profile was isocratic 0–3 min: 5 % B, 3 → 10 min: 100 % B, 10 → 15min: 100 % B, 15 → 17 min: 5 % B (curve 1). The patient LC profile was isocratic 0–5 min: 5 % B, 5 → 28 min: 100 % B, 28 → 38 min: 100 % B, 38 → 45 min: 5 % B (curve 1).

The MS source parameters were as follows: source voltage 4200 V, capillary voltage 35 V, tube lens 100 V, source temperature 275 °C, desolvation temperature 550 °C, sheath gas flow 20 L h^−1^, and auxiliary gas flow 15 L h^−1^. In the full scan mode, the data was acquired between *m/z* 50 and 1500 using a resolution of 30,000 FWHM at *m/z* 400. In data-dependent acquisition mode, MS full scans were followed by three MS/MS scans performed on the first most intense ion from the first scan event, without dynamic exclusion. Selected precursor ions were fragmented at three different activation types (CID, and HCD at two normalized collision energy) and with a selection window size of 1.0 Th and were recorded in centroid mode. For CID-35, the following settings were used: minimum signal required 20′000, isolation width 5, normalized collision energy 35, activation Q 0.25, and activation time 30 ms. For HCD-35, the following settings were used: minimum signal required 10′000, isolation width 5, normalized collision energy 35, and activation time 30 ms. Finally, for HCD-50, the following settings were used: minimum signal required 10′000, isolation width 5, normalized collision energy 50 and activation time 30 ms. Scan duration for both MS and MS/MS was 1 s. Data acquisition was managed using ThermoFisher® Xcalibur software (version 4.1; ThermoFisher®, San Jose, USA). The following MS precursor *m/z* were rejected (196.17, 214.09, 215.09, 216.08, 391.1, 392.1, 459.17, 461.18, 871.57, 872.57). To increase the probability of obtaining high quality MS/MS spectra of a given drug, no dynamic exclusion was applied.

It is important to note that the chromatographic gradients employed between the creation of the database (which spanned a total duration of 17 min) and the subsequent analysis of the patients (which spanned a total duration of 45 min) differed. The database was created using concentrated pure standards, whereas the patients were analyzed using a longer and higher resolution chromatography, as only the five most intense ions were selected for MS/MS fragmentation in the data-dependent analysis (DDA) mode. The data dependent analysis in three activation modes (CID-35, HCD-35 and HCD-50) for the five most intense ions was completed in approximately 2.8 s per cycle using our Orbitrap instrument. To obtain the highest number of points and the cleanest MS/MS spectra, a 45-min gradient was identified as an optimal compromise for drug screening.

### 2.4 Data pre-processing parameters

Raw data obtained from the LC–MS/MS system were converted from ThermoFisher's proprietary raw format into open source mzXML format using the ProteoWizard tool msconvert (version 3.0.10051, Vanderbilt University, United States) [28]. **(****http://proteowizard.sourceforge.net/****)**. MZmine 2, an open-source project that provides software for the processing, visualization, and analysis of MS-based molecular profiling data, was used [29]. MZmine is a popular open-source, cross-platform MS data processing software with an advanced graphical user interface that visually optimizes parameters and examine the results of each processing step. It also enables the export of a batch file containing all the steps and parameters used in the processing, thereby facilitating its reproducibility. MS and MS/MS spectra were extracted using MZmine 2.51 with a mass detection noise level set to 1E3 and 1E0, respectively. Chromatograms were then generated using the ADAP algorithms [30] with a minimum group size of 4 scans, a group intensity threshold of 1000 and an *m/z* tolerance of 0.005 Da (approximately 15 ppm). We used ADAP Wavelets algorithm for the deconvolution of chromatograms with the following settings: signal-to-noise ratio 3, coefficient/area ratio 1, peak duration range 0.05–1 min, retention time wavelet range 0.00–0.5, *m/z* range for MS/MS scan pairing 0.025, and retention time (tR) range for MS/MS scan pairing 0.1 min. De-isotopization was performed using the Isotope Peak Grouper algorithm with an *m/z* tolerance of 0.005 (*m/z* < 500) and 10 ppm (*m/z* > 500) and a tR tolerance of 0.2 min. The peaks were aligned using the Join Aligner method with the following parameters: *m/z* tolerance 0.005 (*m/z* < 500) and 10 ppm (*m/z* > 500) and absolute tR tolerance 0.2 min. Each MS/MS scan was assigned to the corresponding MS scan with a tR tolerance of 0.2 min and an *m/z* tolerance of 0.005 (*m/z* < 500) and 10 ppm (*m/z* > 500). The peak list was then filled using the “same RT and *m/z* range gap filler” module with an *m/z* tolerance of 0.005 (*m/z* < 500) and 5 ppm (*m/z* > 500). Once data preprocessing and MS 1 annotation using MZmine was done, the molecular network was generated.

### 2.5 Molecular network analysis

The MNs were created, visualized and finally annotated using MetGem 1.2.2 software **(**https://metgem.github.io**)** [31]. Due to the use of HR data, the base parameters were changed to 0.02 *m/z* for the mass tolerance of the precursor and fragment ions used in the MS/MS spectral library search, and to 0.02 *m/z* for the mass tolerance of the fragment ions used in the molecular network. Connections between nodes were created when the cosine score was greater than 0.5 and the minimum number of common fragment ions shared by two MS/MS spectra was 3. Connections between two nodes were maintained in the network only if each node was among the top 10 most similar nodes. Once the network had been created, the MS2 spectra were compared with different GNPS, our local database or a CFM-ID (Competitive Fragmentation Modeling for Metabolite Identification) *in silico* spectral libraries. The library spectra queries were performed with the same parameter values as those defined for the construction of the network, except for the cosine value, which should be greater than 0.2. GNPS libraries included (https://gnps.ucsd.edu/ProteoSAFe/libraries.jsp): NIH Clinical Collection 1, NIH Clinical Collection 2, FDA library Pt 1&2, Sigma's Mass Spectrometry Metabolite Library (MSMLS), and pharmacologically active compounds in the NIH small molecules repository. In order to be as exhaustive as possible, all of these libraries have been consulted for a non-targeted toxicology screening.

### 2.6 MetWork and CFM-ID annotation

To identify individual compounds, the resulting MS/MS spectra, together with the chromatographic retention time of the precursor ion masses of the compound of interest, were (ideally) compared with authentic standard MS/MS spectra and retention times to validate compound identity. In most laboratories, the accessibility of many authentic chemical reference materials is limited. Therefore, the identification of putative metabolites is more commonly carried out. Presumptive identification (MSI level 2) is achieved by comparing MS/MS spectra with experimentally collected reference spectra from various MS/MS spectral databases. Competitive Fragmentation Modeling ID (CFM-ID) accurately predicts electrospray ionization mass spectra (ESI-MS/MS) from chemical structures and helps identify compounds by MS/MS spectral matching [32,33]. CFM-ID combines a group of three input spectra (for ESI spectra, low/10V, medium/20V, and high/40V energy levels).

MetWork [34] is a web application developed to anticipate natural products identification using *in silico* methods for metabolization of known metabolites and fragmentation simulation [35]. Simulation data are compared with experimental LC-MS/MS data with a spectrum similarity score. This score provides an insight that can trigger an assessment method to confirm the annotation of a metabolite of the network with the generated structure [36]. MetWork could therefore accelerate and optimize product analysis by highlighting putative structures in biological matrices. This software is freely available as a web application (https://metwork.pharmacie.parisdescartes.fr) and its code is distributed with the open-source GPL license. Application of this software results in a particularly visible propagation of the annotations on the molecular network, which can be used to reinforce the relevance of the annotations [35]. MetWork annotations were ﬁltered and this file was used for annotation of the previously obtained molecular network and was further visualized with MetGem software.

## 3 Results & discussion

### 3.1 Drug database creation & comparison of fragmentation acquisition

MS/MS product ions are of real importance as drug identification relies on comparison of MS/MS spectra between experimental patient acquisition and the experimental or even *in silico* database. Numbers of identical product ions and similar pattern are important for identification as well as specific product ions which could occur at higher collision energy.

As a first step, a database of 155 standards was built as a local database highlighting the need for a specific database containing drugs commonly used in hospital wards. In this database, parent drugs and some metabolites belong to various clinical categories, such as psychotropic drugs (antidepressants, neuroleptics, benzodiazepines and related Z-drugs), painkillers (opoids including tramadol), cardiovascular drugs (beta-blockers, angiotensin renin blockers, calcium channel blockers, sartans), anti-inflammatory drugs, antiepileptics, antibiotics/antifungals, antiretrovirals, immunosuppressants, oral anticoagulant drugs, and non-commercialized drugs (drugs of abuse) such as cocaine, amphetamine, GHB … The complete list can be found in the supplementary data (Table S1).

For each of the 155 standards, different experimental MS/MS activation acquisitions using CID at 35 V and HCD at 35 and 50 V were recorded to further examine their complementarity for drug annotation once the molecular network is generated. Finally, all 465 recorded MS/MS spectra were uploaded onto GNPS databases, and will, we hope, be helpful for drug identification. These MS/MS spectra are also available as.mgf files in supplementary data to perform local identification.

**Fig. 1:**
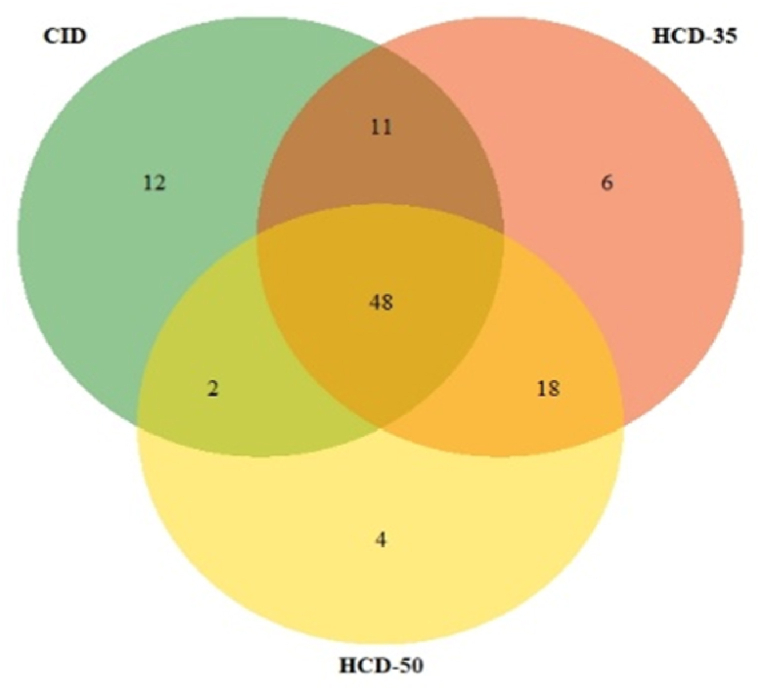
Venn diagram corresponding to the similarity and specificity of the drug identification based on MS/MS spectra acquired at three collision energy values (CID 35, HCD 35 and HCD 50).

As a second step, in addition to the experimental MS/MS study, the *in silico* MS/MS spectra were generated for the same 155 standards. For this purpose, CFM-ID was used through the combination of three energy spectra. *In silico* and experimental spectra were then compared as illustrated using Venn diagrams **(**Fig. 1**)**. Different databases were loaded as MN onto MetGem software, and the generated MN was interrogated using all databases for drug identification. As a starting point, the *in silico* database was interrogated by experimental MN. Over the 155 nodes (drugs), the CID and HCD databases allowed identification of 73, 83 and 72 drugs for CID, HCD-35 and HCD-50, respectively. Going a little bit further, 12 were specific for CID, 6 for HCD-35, and 4 for HCD-50. Forty-eight drugs were found by all libraries and 11 were common for CID-HCD-35, 2 for CID-HCD-50, and 18 for HCD-35/HCD-50. Other comparisons (CID/HCD 35/HDC 50 *vs.* all other databases) were also performed (data not shown). In relation to the 155 drugs of our experimental database, CFM-ID gave comparable results in 50 % of cases whatever the experimental database used.

It is worth noting that each different MS/MS acquisition (using CID or HCD as fragmentation mode) has a specific interest. As an example, low-molecular-weight amphetamines or related amphetamine structures are either well recognized by all activation types or more specifically by CID fragmentation. This may be because low-molecular-weight compounds and high-energy dissociation fragmentation generate too few product ions in the MS/MS spectra for complete identification through *in silico* fragmentation. In contrast, relatively high-molecular-weight compounds with low numbers of fragments, such as buprenorphine or amiodiarone, are more clearly recognized using high-energy dissociation activation types like HCD 35 or 50. Finally, these results highlight the need for different databases for more complete identification and annotation. For the *in silico* database, as CFM-ID is a machine learning technique, a better match could potentially be reached in the near future between the predicted spectra and the input spectra by increasing the number of molecules tested. It should be emphasized that the comparison between experimental and CFM-ID modeled-MS/MS spectra identified more than 50 % of the commercial drugs. As MetWork uses CFM-ID to generate *in silico* MS/MS spectra of drug metabolites, efficient identification using this tool is mandatory.

### 3.2 MetWork: a tool for investigation of metabolites and new drug annotation

During toxicological screening, identification of the parent compound is the key step to make the assessment. Nevertheless, the identification of metabolites can confirm the consumption of a specific drug while increasing the detection window. For instance, cocaine and benzoylecgonine displayed a plasma half-life of 1 and 5 h, respectively, illustrating the importance of metabolites in toxicological screening. Furthermore, drug metabolism reactions in humans are well known and involve, among others, demethylation, oxidation, and conjugation reactions, mostly glucurono- or sulfo-conjugation [37]. Manual inspection of the LC-HRMS data of potential metabolites can help to identify them. Nevertheless, an exhaustive identification by a manual approach is tedious, not very efficient, and requires real technical competence in MS. In view of the predictive aspect of these metabolization reactions, the use of bioinformatics solutions can therefore be considered. Among these solutions, MetWork is a recent online application that allows structure prediction from LC-HRMS data and has proven efficient in the anticipation of natural product structures [38,39].

To complete the proposed strategy to annotate and identify drugs in patients’ plasma, after (i) the need to build a specific database with drugs used in hospitals, (ii) the molecular network generation using MS1 preprocessing, MetWork web application will link drugs and allow identification of metabolites or new drugs. The idea, in this instance, is to start from the known molecules/drugs of the plasma extract and to apply *in silico* different chemical reactions to generate new related compounds.

**Fig. 2:**
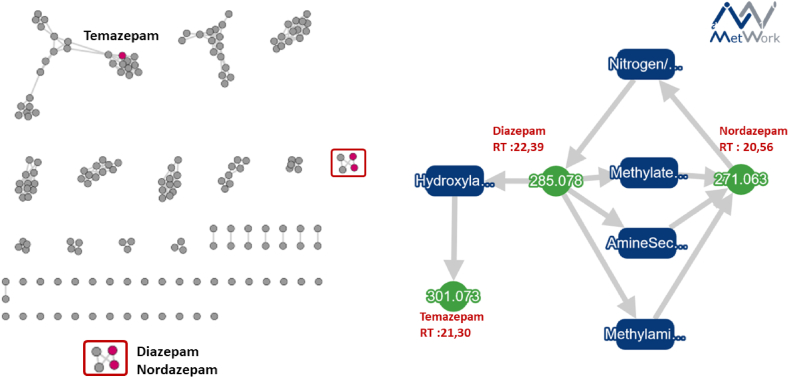
Molecular network-based identification of diazepam metabolites in a patient using MetWork for structure prediction. For the molecular network, solid red dots (red nodes) represent the molecules that have been identified following a query of various databases, with a cosine value exceeding 0.5 and, at least, three identical MS/MS fragments, as mentioned in section 2.5. Conversely, grey dots (grey nodes) represent molecules that are present within the sample but have not been identified. The presence of drugs within the same cluster, which are linked by edges, highlight the fact that these molecules share a certain structural analogy.

In the first example, once MN was generated, diazepam, nordazepam, and temazepam were annotated using the different databases. Physiologically *via* oxidative pathways, phase I metabolism of diazepam leads to the formation of nordazepam (N-desmethyldiazepam) and temazepam (hydroxydiazepam), which are further metabolized to oxazepam. As seen in Fig. 2, once the depth of metabolization parameters and the list of reactions are defined, MetWork linked these metabolites. It is therefore possible to visualize the direct connection between the anchor node (green) and the produced metabolites with the cosine value, demonstrating the degree of confidence of the annotation. As a proof of concept, for the patient, the LC-MS/MS data contained 170 ions of which 3 have known structures, annotated as starting molecules: diazepam, nordazepam, and temazepam. With this sample and the available reactions, we expected to obtain an O- or N-demethylation filiation for these different benzodiazepines. MetWork confirmed this. The same example was studied by annotating a single molecule where several cycles of metabolism were performed, which leads to the same conclusion.

**Fig. 3:**
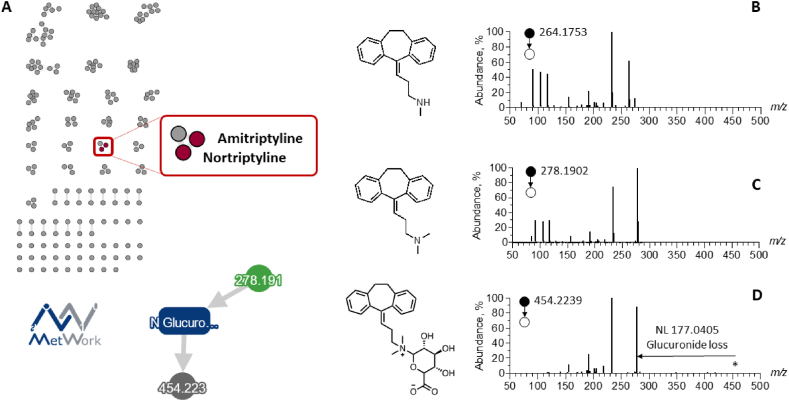
Molecular network-based identification of amitryptiline metabolites in a patient using MetWork web application for structure anticipation. (A) Molecular network of the MS/MS spectra corresponding to the analysis of the patient's plasma extract. MS/MS spectra of –B) amitryptiline and (C) nortriptyline and (D) glucurono-conjugated molecule of nortriptyline. In the figure, the red dot indicates that the compound is identified, while the grey dot indicates that the compound is not annotated. For patients samples MS/MS spectra are measured by CID.

**Fig. 4:**
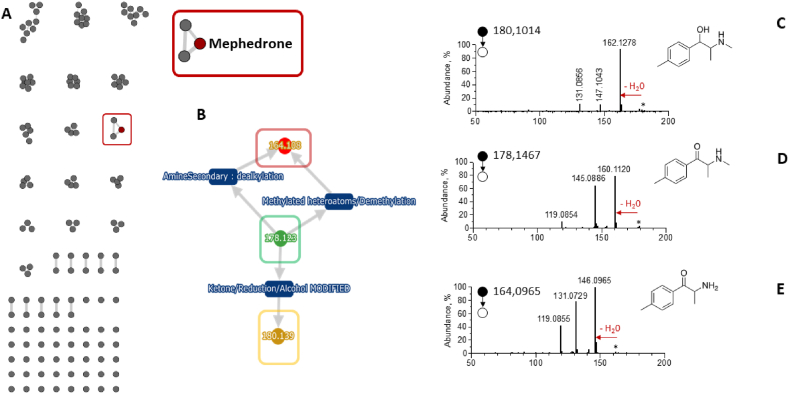
Molecular network of mephedrone and its metabolites obtained using LC-HRMS data of a poisoned patient. (B) scheme of the reaction of MetWork to elucidate major mephedrone metabolites. MS/MS spectra of [M+H]^+^ ion of (C) dehydro-mephedrone, (D) mephedrone, and (E) N-desmethyl mephedrone. The proposed structures of mephedrone metabolites are also in perfect agreement with human metabolism as already published (ref 42).

In a second example, for another patient, the LC-MS/MS data contained 179 ions of which 2 have known structures, annotated as starting molecules: amitriptyline and nortriptyline, as shown in Fig. 3. Phase 2 metabolism was highlighted, as amitriptyline glucuronide was proposed, which is coherent with human metabolic pathways [40]. Behind this annotation, raw MS/MS spectra are shown in figure (3B, C, D) to illustrate the spectral homologies between amitriptyline and its phase I as well as phase II metabolites. The annotation of amitriptyline glucuronide was also supported by manual inspection of its MS/MS spectrum. Indeed, the MS/MS spectrum of the [M+H]^+^ ion at *m/z* 454.2225 displayed the neutral loss of 176.0320, diagnostic of the glucuronide loss (Fig. (4D)). The resulting product ion thus undergone into a similar fragmentation pattern than amitriptyline one, *i.e.* neutral losses of 45.0579 and 87.1051 corresponding to dimethylamine and dimethylbutenamine losses, respectively.

Since elucidating metabolism is a fundamental goal of pharmacological/toxicological studies and, in parallel, visualization tools that allow rapid metabolism exploration are still/currently underutilized, an aim of this proposed strategy is to emphasize the value of molecular networks combined with MetWork for exploring drug metabolism and/or for drug identification. The advantages of such a strategy are that the analysts know which drugs are ingested by patients, as these are commercial drugs. Using this approach, parent drugs and metabolites could be linked, increasing confidence in the results. New metabolites, such as phase I and/or phase II metabolites, could be highlighted, thus giving a better view of human metabolism and providing greater certainty. In addition, another major advantage of finding additional metabolites is that it improves confidence in the identification of a drug. Therefore, even without commercial standards, this annotation could be made, and it could be envisaged that by enriching the database/dataset with these newly annotated compounds the scope of the database would be enlarged. From “plausible”, this annotation becomes a “known” structure and allows annotation propagation, and identification, in the network.

### 3.3 Application of molecular network + MetWork to clinical toxicology screening

#### 3.3.1 Tramadol and its metabolites

**Fig. S1:**
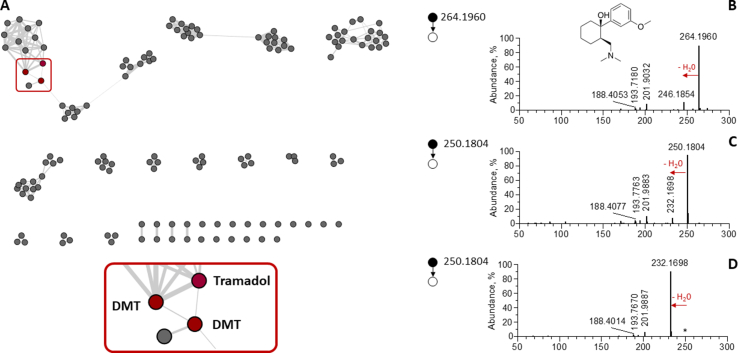
Molecular network-based identification of tramadol metabolites in a patient using MetWork for structure anticipation. (A) Molecular network of the MS/MS spectra corresponding to the analysis of the patient's plasma extract. MS/MS spectra of (B) tramadol, (C) O-desmethyltramadol and (D) N-desmethyltramadol. In the figure, the red dot indicates that the compound is identified, while the grey dot indicates that the compound is not annotated. For patients samples MS/MS spectra are measured by CID.

For this patient, tramadol was found and identified by the reference methods. Using the MN approach, tramadol and its metabolite O-desmethyltramadol were annotated. Two annotations were recorded for desmethyltramadol metabolites at two different retention times. This led to two different isomeric metabolites such as N- and O-desmethyltramadol (ODT and NDT, respectively). Both metabolites are present physiologically, with O-desmethyl being the major active metabolite whilst N-desmethyl is an inactive less abundant metabolite. Nevertheless, based only on the MS point of view, with these uninformative tramadol and metabolites MS/MS spectra resulting in a water loss (−18), it is difficult to assign without ambiguity O- and N- desmethyltramadol metabolites, which share the molecular formula C_15_H_23_NO_2_ and the [M+H]^+^ ion at *m/z* 250.1798, as shown in supplementary data (Fig. S1). Therefore, due to similar molecular mass and fragmentation patterns, LC separation is required in order to be able to distinguish ODT from NDT in MS/MS analysis.

**Fig. S2:**
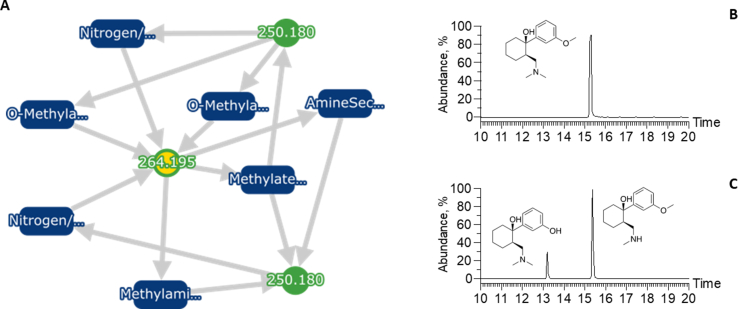
MetWork for structural identification of tramadol metabolites. (A) Scheme of the demethylated reaction operated in MetWork. Extracted ion chromatogram at (B) *m/z* 264.1960 and (C) *m/z* 250.1802 corresponding to the [M+H]^+^ ion of tramadol and desmethyltramadol, respectively. Retention times of tramadol, O-desmethyltramadol, and N-desmethyltramadol were 15.3, 13.2, and 15.36 min, respectively.

High-resolution LC obviates this uncertainty. O-Desmethyltramadol is more polar than its N dealkylated isobaric metabolites and so results in different retention times. For this patient, O-, N-desmethyltramadol, and tramadol eluted at 13.2, 15.36, and 15.30 min respectively, as shown in supplementary data (Fig. S2). As a final proof, all standards were available and separately injected, thus confirming this assumption, as O-, N-desmethyltramadol, and tramadol eluted at 9.19, 9.63 and 9.64 min, respectively.

Finally, increasing confidence in the results, MetWork confirmed the MS/MS spectra homologies between tramadol and its two isobaric metabolites, linking the parent drug to its two metabolites.

#### 3.3.2 Venlafaxine & amitriptyline

For this second example, amitriptyline and venlafaxine were both found in this patient, using both approaches (reference and MN strategies).

**Fig. S3:**
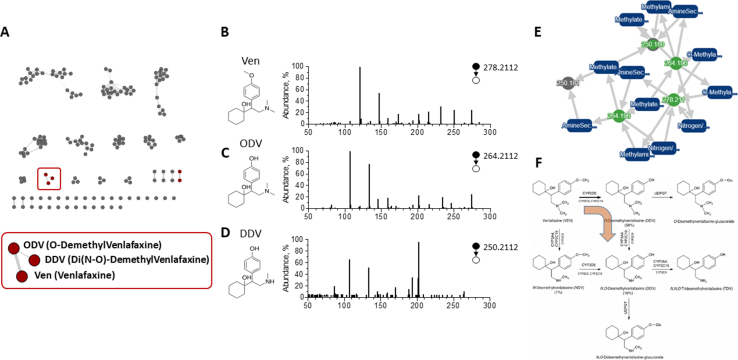
MetWork for structural identification of venlafaxine metabolites. (A) Molecular network of venlafaxine cluster. MS/MS spectra corresponding to the [M+H]^+^ ion of (B) venlafaxine, (C) O-desmethylvenlafaxine, and (D) N,O didesmethylvenlafaxine. (E) Scheme of the demethylated reaction of venlafaxine operated in the MetWork platform. (F) Metabolic pathway of venlafaxine. The arrow indicates the primary metabolic pathways of venlafaxine in humans. The majority of venlafaxine is metabolized to O-desmethylvenlafaxine (ODV), which is then metabolized to didesmethylvenlafaxine (DDV).

Venlafaxine major phase I dealkylated metabolite was also annotated as shown in supplementay data (Fig. S3). MetWork propagated and increased the knowledge and confidence of the result by annotating and linking another phase I metabolite, didesmethylvenlafaxine.

**Fig. S4:**
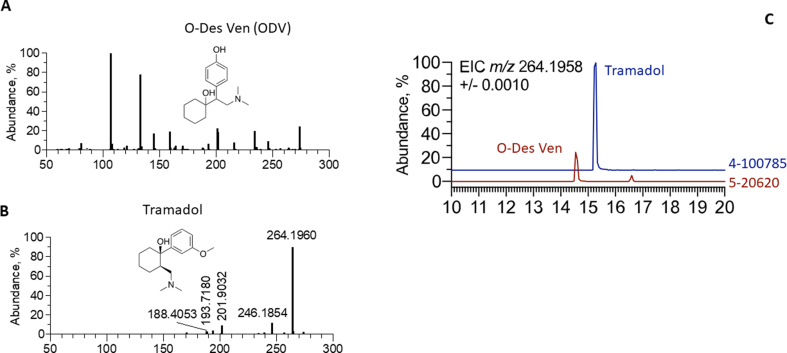
MS/MS spectra of [M+H]^+^ ion of (A) O-desmethylvenlafaxine and (B) tramadol. (C) Extracted ion chromatogram at *m/z* 264.1958 of LC-HRMS data obtained through the analysis of plasma samples of two patients who ingested tramadol and venlafaxine, respectively.

It is important to note that tramadol and desmethylvenlafaxine are isomeric drugs, and unfortunately are often found together in patients who have ingested multiple drugs. Even though the MS/MS spectra of these two drugs are quite different, they could be misannotated in the case of poor-quality MS/MS spectra. An efficient way to confirm the presence of drugs may also be achieved through patient comparisons using MZmine software. Considering patients who have only ingested tramadol or venlafaxine, pairs of exact *m/z* values and retention time flags are used to confirm or discount the presence of drugs (supplementary data Fig. S4).

**Fig. S5:**
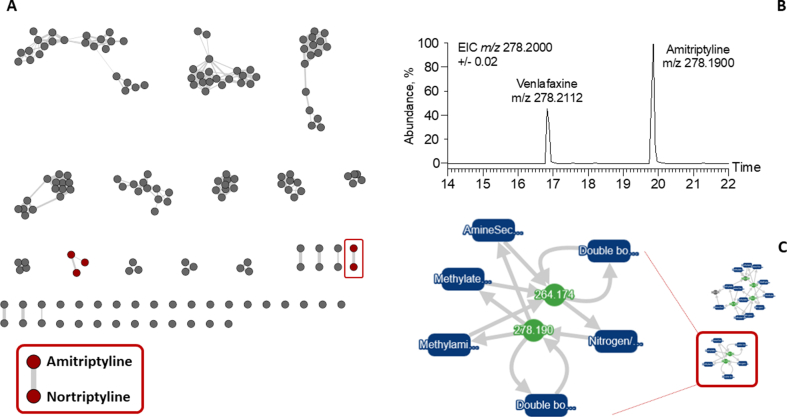
(A) Molecular network of amitryptiline. (B) Extracted ion chromatogram at *m/z* 278.2000 and (C) scheme of the reaction of MetWork.

Amitriptyline and its major metabolite nortriptyline were annotated, and this was confirmed by MetWork linking parent and metabolite, as shown in supplementary data (Fig. S5). Amitriptyline and venlafaxine are also quite close in terms of mass accuracy. As already mentioned, MS/MS spectra as well as retention times provided orthogonal and complementary information leading to the unambiguous identification of both drugs (supplementary data Fig. S5).

#### 3.3.3 Mephedrone and its metabolites

Many new designer drugs have emerged in recent years, such as cathinone derivatives. A common drug is mephedrone (4-methylmetcathinone or 4-MMC). It was discovered in this patient's urine through GC-MS analysis screening [41], whilst, in plasma, the reference methods described earlier did not identify mephedrone. For this patient, MN identified this drug through the *in silico* database. Questions arise about the certainty of these results and MetWork increased confidence in this finding by annotating two phase 1 metabolites, as already described in the literature [42]. N-demethylation was identified as well as a reduction of the keto function leading to 1-dihydromephedrone. *In vitro* studies also suggest that mephedrone metabolism is mediated through the CYP2D6 isoenzyme. Molecular network annotation as well as MS/MS spectra are shown in Fig. 4.

This workflow is highly dependent on the predictive value of CFM-ID and, in this case, it was pleasing to find that the predicted MS2 spectra of our various metabolites were of good quality regardless of their original structures. Fortunately, the cosines of most proposed structures are all greater than 0.5, indicating a high level of confidence. Correct prediction generally occurs with cosine values in the range of 0.3–0.6. These are very good values when dereplicating against *in silico* MS2 spectra [23]. The proposed structures of mephedrone metabolites are also in perfect agreement with human metabolism as already published [42].

### 3.4 Comparison of MN with reference laboratory strategies

Following the database implementation, plasma of hospitalized patients was randomly analyzed using LC-ESI-HRMS in positive ion mode. The data were then processed with a dedicated workflow on MZmine to build an MN as already described in the materials and methods section. The experimental, *in silico*, and available clinical GNPS databases were consulted to identify drugs in these patients. MetWork was used to detect, if possible, the corresponding drug metabolites.

This procedure was performed for 60 hospitalized patients investigated either through reference methods used day-to-day in clinical laboratories or using our LC coupled to high-resolution MS approach. The reference methods ranged from immunoassays to LC diode array MS, GC-MS, and LC-tandem MS, and are briefly described elsewhere [43]. Further description is beyond the scope of this paper.

For 43 out of these 60 patients (72 %), drugs were always found in common using both strategies. For 6 patients (10 %) no drugs were found in plasma using either the reference methods or the molecular network approach. Therefore, similar results were obtained by both approaches for a total of 49 patients out of 60 (82 %).

For 6 patients (10 %), no annotation could be made using the MN approach, whereas drugs were found using the reference methods. Conversely, for 5 patients (8 %), annotations were made using high-resolution MS/MS databases, whereas any medications were highlighted using the reference methods. For these patients, MS/MS spectra were compared with the database to assess the presence of the drug in the sample and avoid false-positive annotation.

Failure to find drugs or metabolites using our molecular approach strategy might be explained because of poor sensitivity and relatively low sample volume (100 μL) and/or as acquisition events were only focused on the 1st most intense ion. For reference methods, sample volumes up to 500 μL could be used along with specific and selective sample preparation to concentrate analytes of interest and maximize their limit of detection. Also, for real patients, potent drugs are not always highly concentrated in plasma and could be surrounded by matrix ions. In our case, this is limited by the use of high-performance LC prior to the detection by MS.

The lack of identification using the reference methods, compared to the molecular network approach using different databases, may be attributed to molecules not usually searched for in cases of poisoning (such as antibiotics) or to inappropriate techniques (labile drugs *vs.* GC-MS analyses, polar drugs such as acetaminophen *vs.* SPE-UPLC-DAD/MS screening).

The issue of detection limits has not been the focus of this study, as the primary objective was to demonstrate the feasibility of identifying the main substances responsible for intoxication and subsequently annotating new metabolites using molecular network coupled to the Metwork web server. The fact that the same incriminating compounds were identified in 72 % of patients, as previously stated, lends support to the potential application of this strategy in a clinical toxicology laboratory. However, before it can be applied daily, the determination of the detection limit will need to be characterized.

Interestingly, similar results were obtained with classical or feature-based MN, thus validating the preprocessing parameters used. Furthermore, feature-based MN reduces redundancy in the resulting preprocessed molecular network, thus simplifying the discovery of drugs and structure-related compounds. MZmine pre-processing also allows the export of a batch file that contains all the steps and parameters used in the processing, thus allowing for its potential to be reproduced. Using the preprocessing strategy, MN is simplified, thus facilitating discovery of structurally related compounds. Nevertheless, skilled analysts are still needed as much information is easily overlooked if the parameters are incorrectly tuned.

These different examples illustrate this powerful tool through phase I or phase II metabolite discovery. These examples also validate the *in silico* findings linking metabolites to their annotated parent drugs. Phase 1 and 2 metabolites, once MS/MS spectra are validated, could be added to the database and thus enlarge the scope of molecules potentially found, even without standards. Besides these experimental annotations, the molecular network has been enriched with computer-generated metabolites using MetWork. These *in silico* generated compounds were obtained *via* various reaction steps, consistent with physiological biochemistry, including, N- and O-demethylation, as well as N-oxidation.

These results and their application in clinical toxicology and therapeutic drug monitoring are very promising. However, as expected, MN should be interpreted in the light of several limitations. It requires optimization at each stage of data processing and a strong background in LC-MS/MS analysis.

As only a positive ionization process has been conducted, it is important to consider that analytes that undergo ionization exclusively in the negative electrospray mode will not be detected with the current methodology. Also, like all screening processes, it is likely to produce false positive results. For this reason, the analyst responsible for validating the results will need to compare them with the patient's clinical situation. However, a number of safeguards are in place to help validate the results:. i) High Resolution chromatography for 45 min to elute the various compounds present in the patient's plasma, ii). Reprocessing of the data using MZmine to deconvolute the results (to take account of the isotopic contribution, for example) and obtain a matrix in which one line corresponds to one compound. Identification is also carried out by comparing spectra with reference spectra, and a positive result is only returned for a cosine above a given threshold. Finally, a MetWork query will also be used to link different compounds together (phase 1 and/or 2 metabolites and parent substance).

The possibility that other metabolites, especially phase II metabolites, could have been visualized with different LC-MS/MS settings cannot be excluded. MetWork will greatly facilitate the work of scientists involved in metabolite identification. However, analytical expertise is critical to achieving quality results. First, the MS/MS data must be of good quality to be exploitable. Then, the starting (anchoring) molecules must be correctly identified, by searching in databases of mass spectra of known molecules. In fact, for some patients, despite the correct computer-generated structures of tramadol and its metabolites, mis-annotations are indicative of the limitations of MetWork's MS/MS spectra prediction module, notably for patients who have ingested venlafaxine, amitriptyline, and tramadol. This will lead to mis-annotations and to erroneous interpretations, since the metabolic reactions are identical. Choice of reactions is important in finding relevant structures: the more reactions chosen, the greater the chance of finding the right one, though this generates more calculations. A good knowledge of the reactions appropriate to the species studied or its family can help in this choice. In this context, more work is needed to improve the accuracy of MS/MS spectra prediction algorithms by, for example, introducing a rule-based fragmentation strategy (CFM-ID 3.0) [33]. Practical knowledge of the relevant thresholds (cosine, mass tolerance) is also required. Finally, it is up to the analyst to validate the proposed structures with the appropriate means.

A limitation of the reliable use of MetWork is the quality of the *in silico* fragmentation. Different tools exist and are of varying quality. Strictly speaking, there is not one tool that is better than the others. Each has its specialties, and each specific one will be relevant for certain types of structures only. For MetWork, the choice was made to use CFM-ID. Other solutions can be tested subsequently. Nevertheless, CFM-ID is a very efficient method, and uses machine learning tools. These methods are based on learnings from experimental fragmentation data of known molecules. We can therefore expect more and more reliable predictions as new experimental data become available. As already mentioned, there was a 50 % recovery between the CFM-ID *in silico* database and our experimental CID and HCD databases, which is a good starting basis. As CFM-ID is a machine learning technique, a better match will potentially be reached between the predicted spectra and the input spectra by increasing the number of molecules tested. Due to its machine learning function, CFM-ID gives uneven performances across structural series. Structures close to those in the training set of CFM-ID therefore have a greater chance of correct prediction of their tandem mass spectrum than structures less close. Nevertheless, even without reference standards, annotations and suggestions could be validated and therefore updated in the database.

## 4 Conclusion

In conclusion, we propose to use LC-HRMS combined with the MetWork webserver using a molecular network approach in clinical pharmacology/toxicology. Proof of concept of this strategy was discussed and hopefully validated through different steps (experimental database vs. *in silico* database), in a 60-patient retrospective study. Our first aim was to identify drugs present in hospitalized patients and our second aim was potential identification of new metabolites or new drugs. This approach used in pharmacology could allow annotation of new metabolites from old drugs and identification of different therapeutic drugs, whilst in toxicology, new drugs on the market, new metabolites from new designer drugs or drugs of abuse could be “discovered”. This could help clinicians better understand drug-drug interactions and explain toxicity or failure of patient treatments when used in combination with CFM-ID and MetWork to extend the annotation of new drug candidates or old drug metabolites.

## Data availability statement

Data will be made available on request. However, compound experimental MS/MS spectra obtained in CID 35 eV, HCD 35eV and HCD 50 eV were uploaded onto the GNPS “all GNPS Library Spectra” public spectral libraries(https://gnps.ucsd.edu/ProteoSAFe/libraries.jsp). Furthermore, MS/MS spectra acquired under CID 35, HCD 35 and HCD 50 are fully available as local files in supplementary data (.mgf files).

## CRediT authorship contribution statement

**Romain Magny:** Writing – original draft, Visualization, Formal analysis, Data curation. **Yann Beauxis:** Writing – review & editing, Software. **Gregory Genta-Jouve:** Writing – review & editing, Software, Conceptualization. **Emmanuel Bourgogne:** Writing – review & editing, Writing – original draft, Supervision, Formal analysis, Conceptualization.

## Declaration of competing interest

The authors declare that they have no known competing financial interests or personal relationships that could have appeared to influence the work reported in this paper.

## Appendix A Supplementary data

Table S1 (supplementary material) List of 155 common drugs and drugs of abuse often found in patient hospitalized in critical care unit. The table lists the compounds, their molecular weight, their structures in smiles and inchi, as well as their pubchem and CAS number in our experimental/*in silico* database. These drugs belong to commonly prescribed therapeutic classes such as antiepileptics, anti-infective, hypnotics, cardiotropics, antalgic drugs or classical drugs of abuse (like amphetamines, cocaine and metabolites, THC). Table S1 (supplementary material)
