# Supplementary material for: Application of a molecular networking approach using LC-HRMS combined with the MetWork webserver for clinical and forensic toxicology
Source: Heliyon. 2024 Aug 31;10(17):e36735. doi: 10.1016/j.heliyon.2024.e36735 (PMC11402778; doi:10.1016/j.heliyon.2024.e36735)
Supplement: Table S1 (supplementary material) — List of 155 common drugs and drugs of abuse often found in patient hospitalized in critical care unit. The table lists the compounds, their molecular weight, their structures in smiles and inchi, as well as their pubchem and CAS number in our experimental/in silico database. These drugs belong to commonly prescribed therapeutic classes such as antiepileptics, anti-infective, hypnotics, cardiotropics, antalgic drugs or classical drugs of abuse (like amphetamines, cocaine and metabolites, THC). [file mmc1.docx]

| **COMPOUND**  **NAME** | **MOLECULE MASS** | **SMILES (structure)** | **INCHI (structure)** | **PUBMED number** | **CAS number** |
| --- | --- | --- | --- | --- | --- |
| **1-(4-methoxyphenyl)-2-(methylamino)propan-1-one (Methedrone)** | 194,1172787 | CC(C(=O)C1=CC=C(C=C1)OC)NC | InChI=1S/C11H15NO2/c1-8(12-2)11(13)9-4-6-10(14-3)7-5-9/h4-8,12H,1-3H3 | 216281 | 530-54-1 |
| **11-Hydroxytetrahydrocannabinol** | 331,2264948 | CCCCCC1=CC(=C2C3C=C(CCC3C(OC2=C1)(C)C)CO)O | InChI=1S/C21H30O3/c1-4-5-6-7-14-11-18(23)20-16-10-15(13-22)8-9-17(16)21(2,3)24-19(20)12-14/h10-12,16-17,22-23H,4-9,13H2,1-3H3/t16-,17-/m1/s1 | 644022 | 36557-05-8 |
| **14530-33-7 (Alpha-PVP)** | 232,1693143 | CCCC(C(=O)C1=CC=CC=C1)N2CCCC2 | InChI=1S/C15H21NO/c1-2-8-14(16-11-6-7-12-16)15(17)13-9-4-3-5-10-13/h3-5,9-10,14H,2,6-8,11-12H2,1H3 | 11148955 | 14530-33-7 |
| **2-Oxo-3-hydroxy-LSD** | 356,1965917 | CCN(CC)C(=O)C1CN(C2CC3(C4=C(C2=C1)C=CC=C4NC3=O)O)C | InChI=1S/C20H25N3O3/c1-4-23(5-2)18(24)12-9-14-13-7-6-8-15-17(13)20(26,19(25)21-15)10-16(14)22(3)11-12/h6-9,12,16,26H,4-5,10-11H2,1-3H3,(H,21,25)/t12-,16-,20?/m1/s1 | 10155149 | 111295-09-1 |
| **4-Fluoroamphetamine** | 154,1023776 | CC(CC1=CC=C(C=C1)F)N | InChI=1S/C9H12FN/c1-7(11)6-8-2-4-9(10)5-3-8/h2-5,7H,6,11H2,1H3 | 9986 | 459-02-9 |
| **4-Hydroxybutanoic acid (ghb)** | 105,0543441 | C(CC(=O)O)CO | InChI=1S/C4H8O3/c5-3-1-2-4(6)7/h5H,1-3H2,(H,6,7) | 10413 | 591-81-1 |
| **4-Hydroxymidazolam** | 342,0801179 | CC1=NC=C2N1C3=C(C=C(C=C3)Cl)C(=NC2O)C4=CC=CC=C4F | InChI=1S/C18H13ClFN3O/c1-10-21-9-16-18(24)22-17(12-4-2-3-5-14(12)20)13-8-11(19)6-7-15(13)23(10)16/h2-9,18,24H,1H3 | 124449 | 59468-90-5 |
| **56354-06-4 (THC-COOH)** | 345,2057594 | CCCCCC1=CC(=C2C3C=C(CCC3C(OC2=C1)(C)C)C(=O)O)O | InChI=1S/C21H28O4/c1-4-5-6-7-13-10-17(22)19-15-12-14(20(23)24)8-9-16(15)21(2,3)25-18(19)11-13/h10-12,15-16,22H,4-9H2,1-3H3,(H,23,24)/t15-,16-/m1/s1 | 108207 | 56354-06-4 |
| **5747-48-8 (Norquétiapine = N-desalkylQuetiapine)** | 296,1213187 | C1CN(CCN1)C2=NC3=CC=CC=C3SC4=CC=CC=C42 | InChI=1S/C17H17N3S/c1-3-7-15-13(5-1)17(20-11-9-18-10-12-20)19-14-6-2-4-8-16(14)21-15/h1-8,18H,9-12H2 | 11369918 | 5747-48-8 |
| **6-Monoacetylmorphine** | 328,1540582 | CC(=O)OC1C=CC2C3CC4=C5C2(C1OC5=C(C=C4)O)CCN3C | InChI=1S/C19H21NO4/c1-10(21)23-15-6-4-12-13-9-11-3-5-14(22)17-16(11)19(12,18(15)24-17)7-8-20(13)2/h3-6,12-13,15,18,22H,7-9H2,1-2H3 | 520352 | 2784-73-8 |
| **7-Aminoflunitrazepam** | 284,1190903 | CN1C(=O)CN=C(C2=C1C=CC(=C2)N)C3=CC=CC=C3F | InChI=1S/C16H14FN3O/c1-20-14-7-6-10(18)8-12(14)16(19-9-15(20)21)11-4-2-3-5-13(11)17/h2-8H,9,18H2,1H3 | 92294 | 34084-50-9 |
| **7-Hydroxy Quetiapine** | 400,1686629 | C1CN(CCN1CCOCCO)C2=NC3=C(C=C(C=C3)O)SC4=CC=CC=C42 | InChI=1S/C21H25N3O3S/c25-12-14-27-13-11-23-7-9-24(10-8-23)21-17-3-1-2-4-19(17)28-20-15-16(26)5-6-18(20)22-21/h1-6,15,25-26H,7-14H2 | 132203 | 139079-39-3 |
| **abacavir** | 287,1612092 | C1CC1NC2=C3C(=NC(=N2)N)N(C=N3)C4CC(C=C4)CO | InChI=1S/C14H18N6O/c15-14-18-12(17-9-2-3-9)11-13(19-14)20(7-16-11)10-4-1-8(5-10)6-21/h1,4,7-10,21H,2-3,5-6H2,(H3,15,17,18,19)/t8-,10+/m1/s1 | 441300 | 136470-78-5 |
| **acebutolol** | 337,2119074 | CCCC(=O)NC1=CC(=C(C=C1)OCC(CNC(C)C)O)C(=O)C | InChI=1S/C18H28N2O4/c1-5-6-18(23)20-14-7-8-17(16(9-14)13(4)21)24-11-15(22)10-19-12(2)3/h7-9,12,15,19,22H,5-6,10-11H2,1-4H3,(H,20,23) | 1978 | 37517-30-9 |
| **aconitine** | 646,3219113 | CCN1CC2(C(CC(C34C2C(C(C31)C5(C6C4CC(C6OC(=O)C7=CC=CC=C7)(C(C5O)OC)O)OC(=O)C)OC)OC)O)COC | InChI=1S/C34H47NO11/c1-7-35-15-31(16-41-3)20(37)13-21(42-4)33-19-14-32(40)28(45-30(39)18-11-9-8-10-12-18)22(19)34(46-17(2)36,27(38)29(32)44-6)23(26(33)35)24(43-5)25(31)33/h8-12,19-29,37-38,40H,7,13-16H2,1-6H3/t19-,20-,21+,22-,23+,24+,25-,26?,27+,28-,29+,31+,32-,33+,34-/m1/s1 | 245005 | 302-27-2 |
| **acyclovir** | 226,0931892 | C1=NC2=C(N1COCCO)N=C(NC2=O)N | InChI=1S/C8H11N5O3/c9-8-11-6-5(7(15)12-8)10-3-13(6)4-16-2-1-14/h3,14H,1-2,4H2,(H3,9,11,12,15) | 135398513 | 59277-89-3 |
| **alprazolam** | 309,0898741 | CC1=NN=C2N1C3=C(C=C(C=C3)Cl)C(=NC2)C4=CC=CC=C4 | InChI=1S/C17H13ClN4/c1-11-20-21-16-10-19-17(12-5-3-2-4-6-12)14-9-13(18)7-8-15(14)22(11)16/h2-9H,10H2,1H3 | 2118 | 28981-97-7 |
| **amiodarone** | 646,03069 | CCCCC1=C(C2=CC=CC=C2O1)C(=O)C3=CC(=C(C(=C3)I)OCCN(CC)CC)I | InChI=1S/C25H29I2NO3/c1-4-7-11-22-23(18-10-8-9-12-21(18)31-22)24(29)17-15-19(26)25(20(27)16-17)30-14-13-28(5-2)6-3/h8-10,12,15-16H,4-7,11,13-14H2,1-3H3 | 2157 | 1951-25-3 |
| **amitriptyline** | 278,1900498 | CN(C)CCC=C1C2=CC=CC=C2CCC3=CC=CC=C31 | InChI=1S/C20H23N/c1-21(2)15-7-12-20-18-10-5-3-8-16(18)13-14-17-9-4-6-11-19(17)20/h3-6,8-12H,7,13-15H2,1-2H3 | 2160 | 50-48-6 |
| **amoxapine** | 314,1051898 | C1CN(CCN1)C2=NC3=CC=CC=C3OC4=C2C=C(C=C4)Cl | InChI=1S/C17H16ClN3O/c18-12-5-6-15-13(11-12)17(21-9-7-19-8-10-21)20-14-3-1-2-4-16(14)22-15/h1-6,11,19H,7-10H2 | 2170 | 14028-44-5 |
| **amoxicillin** | 366,1115419 | CC1(C(N2C(S1)C(C2=O)NC(=O)C(C3=CC=C(C=C3)O)N)C(=O)O)C | InChI=1S/C16H19N3O5S/c1-16(2)11(15(23)24)19-13(22)10(14(19)25-16)18-12(21)9(17)7-3-5-8(20)6-4-7/h3-6,9-11,14,20H,17H2,1-2H3,(H,18,21)(H,23,24)/t9-,10-,11+,14-/m1/s1 | 33613 | 26787-78-0 |
| **amphetamine** | 136,1117994 | CC(CC1=CC=CC=C1)N | InChI=1S/C9H13N/c1-8(10)7-9-5-3-2-4-6-9/h2-6,8H,7,10H2,1H3 | 3007 | 300-62-9 |
| **apixaban** | 460,1976543 | COC1=CC=C(C=C1)N2C3=C(CCN(C3=O)C4=CC=C(C=C4)N5CCCCC5=O)C(=N2)C(=O)N | InChI=1S/C25H25N5O4/c1-34-19-11-9-18(10-12-19)30-23-20(22(27-30)24(26)32)13-15-29(25(23)33)17-7-5-16(6-8-17)28-14-3-2-4-21(28)31/h5-12H,2-4,13-15H2,1H3,(H2,26,32) | 10182969 | 503612-47-3 |
| **atenolol** | 267,1700426 | CC(C)NCC(COC1=CC=C(C=C1)CC(=O)N)O | InChI=1S/C14H22N2O3/c1-10(2)16-8-12(17)9-19-13-5-3-11(4-6-13)7-14(15)18/h3-6,10,12,16-17H,7-9H2,1-2H3,(H2,15,18) | 2249 | 29122-68-7 |
| **benzoylecgonine** | 290,1384081 | CN1C2CCC1C(C(C2)OC(=O)C3=CC=CC=C3)C(=O)O | InChI=1S/C16H19NO4/c1-17-11-7-8-12(17)14(15(18)19)13(9-11)21-16(20)10-5-3-2-4-6-10/h2-6,11-14H,7-9H2,1H3,(H,18,19)/t11-,12+,13-,14+/m0/s1 | 448223 | 519-09-5 |
| **Bisdesethylchloroquine** | 264,1259253 | CC(CCCN)NC1=C2C=CC(=CC2=NC=C1)Cl | InChI=1S/C14H18ClN3/c1-10(3-2-7-16)18-13-6-8-17-14-9-11(15)4-5-12(13)14/h4-6,8-10H,2-3,7,16H2,1H3,(H,17,18) | 122672 | 4298-14-0 |
| **bisoprolol** | 326,2323085 | CC(C)NCC(COC1=CC=C(C=C1)COCCOC(C)C)O | InChI=1S/C18H31NO4/c1-14(2)19-11-17(20)13-23-18-7-5-16(6-8-18)12-21-9-10-22-15(3)4/h5-8,14-15,17,19-20H,9-13H2,1-4H3 | 2405 | 66722-44-9 |
| **bromazepam** | 316,00772 | C1C(=O)NC2=C(C=C(C=C2)Br)C(=N1)C3=CC=CC=N3 | InChI=1S/C14H10BrN3O/c15-9-4-5-11-10(7-9)14(17-8-13(19)18-11)12-3-1-2-6-16-12/h1-7H,8H2,(H,18,19) | 2441 | 1812-30-2 |
| **bupivacaine** | 289,2271635 | CCCCN1CCCCC1C(=O)NC2=C(C=CC=C2C)C | InChI=1S/C18H28N2O/c1-4-5-12-20-13-7-6-11-16(20)18(21)19-17-14(2)9-8-10-15(17)3/h8-10,16H,4-7,11-13H2,1-3H3,(H,19,21) | 2474 | 38396-39-3 |
| **buprenorphine** | 468,3105588 | CC(C)(C)C(C)(C1CC23CCC1(C4C25CCN(C3CC6=C5C(=C(C=C6)O)O4)CC7CC7)OC)O | InChI=1S/C29H41NO4/c1-25(2,3)26(4,32)20-15-27-10-11-29(20,33-5)24-28(27)12-13-30(16-17-6-7-17)21(27)14-18-8-9-19(31)23(34-24)22(18)28/h8-9,17,20-21,24,31-32H,6-7,10-16H2,1-5H3/t20-,21-,24-,26+,27-,28+,29-/m1/s1 | 644073 | 52485-79-7 |
| **Butylone (bk_MBDB)** | 222,1121934 | CCC(C(=O)C1=CC2=C(C=C1)OCO2)NC | InChI=1S/C12H15NO3/c1-3-9(13-2)12(14)8-4-5-10-11(6-8)16-7-15-10/h4-6,9,13H,3,7H2,1-2H3 | 56843046 | 17762-90-2 |
| **caffeine** | 195,0873756 | CN1C=NC2=C1C(=O)N(C(=O)N2C)C | InChI=1S/C8H10N4O2/c1-10-4-9-6-5(10)7(13)12(3)8(14)11(6)2/h4H,1-3H3 | 2519 | 58-08-2 |
| **carbamazepine** | 237,101963 | C1=CC=C2C(=C1)C=CC3=CC=CC=C3N2C(=O)N | InChI=1S/C15H12N2O/c16-15(18)17-13-7-3-1-5-11(13)9-10-12-6-2-4-8-14(12)17/h1-10H,(H2,16,18) | 2554 | 298-46-4 |
| **carisoprodol** | 261,1806073 | CCCC(C)(COC(=O)N)COC(=O)NC(C)C | InChI=1S/C12H24N2O4/c1-5-6-12(4,7-17-10(13)15)8-18-11(16)14-9(2)3/h9H,5-8H2,1-4H3,(H2,13,15)(H,14,16) | 2576 | 78-44-4 |
| **Cathine** | 152,106714 | CC(C(C1=CC=CC=C1)O)N | InChI=1S/C9H13NO/c1-7(10)9(11)8-5-3-2-4-6-8/h2-7,9,11H,10H2,1H3/t7-,9+/m0/s1 | 441457 | 492-39-7 |
| **cefotaxime** | 456,0639403 | CC(=O)OCC1=C(N2C(C(C2=O)NC(=O)C(=NOC)C3=CSC(=N3)N)SC1)C(=O)O | InChI=1S/C16H17N5O7S2/c1-6(22)28-3-7-4-29-14-10(13(24)21(14)11(7)15(25)26)19-12(23)9(20-27-2)8-5-30-16(17)18-8/h5,10,14H,3-4H2,1-2H3,(H2,17,18)(H,19,23)(H,25,26)/b20-9-/t10-,14-/m1/s1 | 5742673 | 60846-21-1 |
| **celiprolol** | 380,2541066 | CCN(CC)C(=O)NC1=CC(=C(C=C1)OCC(CNC(C)(C)C)O)C(=O)C | InChI=1S/C20H33N3O4/c1-7-23(8-2)19(26)22-15-9-10-18(17(11-15)14(3)24)27-13-16(25)12-21-20(4,5)6/h9-11,16,21,25H,7-8,12-13H2,1-6H3,(H,22,26) | 2663 | 56980-93-9 |
| **chlorpromazine** | 319,1027475 | CN(C)CCCN1C2=CC=CC=C2SC3=C1C=C(C=C3)Cl | InChI=1S/C17H19ClN2S/c1-19(2)10-5-11-20-14-6-3-4-7-16(14)21-17-9-8-13(18)12-15(17)20/h3-4,6-9,12H,5,10-11H2,1-2H3 | 2726 | 50-53-3 |
| **Cibenzoline** | 263,1539986 | C1CN=C(N1)C2CC2(C3=CC=CC=C3)C4=CC=CC=C4 | InChI=1S/C18H18N2/c1-3-7-14(8-4-1)18(15-9-5-2-6-10-15)13-16(18)17-19-11-12-20-17/h1-10,16H,11-13H2,(H,19,20) | 2747 | 53267-01-9 |
| **ciprofloxacin** | 332,1402196 | C1CC1N2C=C(C(=O)C3=CC(=C(C=C32)N4CCNCC4)F)C(=O)O | InChI=1S/C17H18FN3O3/c18-13-7-11-14(8-15(13)20-5-3-19-4-6-20)21(10-1-2-10)9-12(16(11)22)17(23)24/h7-10,19H,1-6H2,(H,23,24) | 2764 | 85721-33-1 |
| **citalopram** | 325,1707915 | CN(C)CCCC1(C2=C(CO1)C=C(C=C2)C#N)C3=CC=C(C=C3)F | InChI=1S/C20H21FN2O/c1-23(2)11-3-10-20(17-5-7-18(21)8-6-17)19-9-4-15(13-22)12-16(19)14-24-20/h4-9,12H,3,10-11,14H2,1-2H3 | 2771 | 59729-33-8 |
| **clomipramine** | 315,1619764 | CN(C)CCCN1C2=CC=CC=C2CCC3=C1C=C(C=C3)Cl | InChI=1S/C19H23ClN2/c1-21(2)12-5-13-22-18-7-4-3-6-15(18)8-9-16-10-11-17(20)14-19(16)22/h3-4,6-7,10-11,14H,5,8-9,12-13H2,1-2H3 | 2801 | 303-49-1 |
| **clonazepam** | 316,0480689 | C1C(=O)NC2=C(C=C(C=C2)[N+](=O)[O-])C(=N1)C3=CC=CC=C3Cl | InChI=1S/C15H10ClN3O3/c16-12-4-2-1-3-10(12)15-11-7-9(19(21)22)5-6-13(11)18-14(20)8-17-15/h1-7H,8H2,(H,18,20) | 2802 | 1622-61-3 |
| **cocaine** | 304,1540582 | CN1C2CCC1C(C(C2)OC(=O)C3=CC=CC=C3)C(=O)OC | InChI=1S/C17H21NO4/c1-18-12-8-9-13(18)15(17(20)21-2)14(10-12)22-16(19)11-6-4-3-5-7-11/h3-7,12-15H,8-10H2,1-2H3/t12-,13+,14-,15+/m0/s1 | 446220 | 50-36-2 |
| **codeine** | 300,1591435 | CN1CCC23C4C1CC5=C2C(=C(C=C5)OC)OC3C(C=C4)O | InChI=1S/C18H21NO3/c1-19-8-7-18-11-4-5-13(20)17(18)22-16-14(21-2)6-3-10(15(16)18)9-12(11)19/h3-6,11-13,17,20H,7-9H2,1-2H3/t11-,12+,13-,17-,18-/m0/s1 | 5284371 | 76-57-3 |
| **colchicine** | 400,1751875 | CC(=O)NC1CCC2=CC(=C(C(=C2C3=CC=C(C(=O)C=C13)OC)OC)OC)OC | InChI=1S/C22H25NO6/c1-12(24)23-16-8-6-13-10-19(27-3)21(28-4)22(29-5)20(13)14-7-9-18(26-2)17(25)11-15(14)16/h7,9-11,16H,6,8H2,1-5H3,(H,23,24)/t16-/m0/s1 | 6167 | 64-86-8 |
| **Cyamemazine** | 324,1526189 | CC(CN1C2=CC=CC=C2SC3=C1C=C(C=C3)C#N)CN(C)C | InChI=1S/C19H21N3S/c1-14(12-21(2)3)13-22-16-6-4-5-7-18(16)23-19-9-8-15(11-20)10-17(19)22/h4-10,14H,12-13H2,1-3H3 | 62865 | 3546-03-0 |
| **Dabigatran** | 472,2088877 | CN1C2=C(C=C(C=C2)C(=O)N(CCC(=O)O)C3=CC=CC=N3)N=C1CNC4=CC=C(C=C4)C(=N)N | InChI=1S/C25H25N7O3/c1-31-20-10-7-17(25(35)32(13-11-23(33)34)21-4-2-3-12-28-21)14-19(20)30-22(31)15-29-18-8-5-16(6-9-18)24(26)27/h2-10,12,14,29H,11,13,15H2,1H3,(H3,26,27)(H,33,34) | 216210 | 211914-51-1 |
| **Desethylchloroquine** | 292,1572254 | CCNCCCC(C)NC1=C2C=CC(=CC2=NC=C1)Cl | InChI=1S/C16H22ClN3/c1-3-18-9-4-5-12(2)20-15-8-10-19-16-11-13(17)6-7-14(15)16/h6-8,10-12,18H,3-5,9H2,1-2H3,(H,19,20) | 95478 | 1476-52-4 |
| **desipramine** | 267,1852987 | CNCCCN1C2=CC=CC=C2CCC3=CC=CC=C31 | InChI=1S/C18H22N2/c1-19-13-6-14-20-17-9-4-2-7-15(17)11-12-16-8-3-5-10-18(16)20/h2-5,7-10,19H,6,11-14H2,1H3 | 2995 | 50-47-5 |
| **Desmethylclomipramine (norclomipramine)** | 301,1463264 | CNCCCN1C2=CC=CC=C2CCC3=C1C=C(C=C3)Cl | InChI=1S/C18H21ClN2/c1-20-11-4-12-21-17-6-3-2-5-14(17)7-8-15-9-10-16(19)13-18(15)21/h2-3,5-6,9-10,13,20H,4,7-8,11-12H2,1H3 | 622606 | 303-48-0 |
| **Desmethylflunitrazepam** | 300,0776194 | C1C(=O)NC2=C(C=C(C=C2)[N+](=O)[O-])C(=N1)C3=CC=CC=C3F | InChI=1S/C15H10FN3O3/c16-12-4-2-1-3-10(12)15-11-7-9(19(21)22)5-6-13(11)18-14(20)8-17-15/h1-7H,8H2,(H,18,20) | 520217 | 2558-30-7 |
| **Diacetolol** | 309,1806073 | CC(C)NCC(COC1=C(C=C(C=C1)NC(=O)C)C(=O)C)O | InChI=1S/C16H24N2O4/c1-10(2)17-8-14(21)9-22-16-6-5-13(18-12(4)20)7-15(16)11(3)19/h5-7,10,14,17,21H,8-9H2,1-4H3,(H,18,20) | 50894 | 22568-64-5 |
| **diazepam** | 285,0786407 | CN1C(=O)CN=C(C2=C1C=CC(=C2)Cl)C3=CC=CC=C3 | InChI=1S/C16H13ClN2O/c1-19-14-8-7-12(17)9-13(14)16(18-10-15(19)20)11-5-3-2-4-6-11/h2-9H,10H2,1H3 | 3016 | 439-14-5 |
| **DIHYDROCODEINE** | 302,1747936 | CN1CCC23C4C1CC5=C2C(=C(C=C5)OC)OC3C(CC4)O | InChI=1S/C18H23NO3/c1-19-8-7-18-11-4-5-13(20)17(18)22-16-14(21-2)6-3-10(15(16)18)9-12(11)19/h3,6,11-13,17,20H,4-5,7-9H2,1-2H3/t11-,12+,13-,17-,18-/m0/s1 | 5284543 | 125-28-0 |
| **diltiazem** | 415,1683285 | CC(=O)OC1C(SC2=CC=CC=C2N(C1=O)CCN(C)C)C3=CC=C(C=C3)OC | InChI=1S/C22H26N2O4S/c1-15(25)28-20-21(16-9-11-17(27-4)12-10-16)29-19-8-6-5-7-18(19)24(22(20)26)14-13-23(2)3/h5-12,20-21H,13-14H2,1-4H3/t20-,21+/m1/s1 | 39186 | 42399-41-7 |
| **domperidone** | 426,1688527 | C1CN(CCC1N2C3=C(C=C(C=C3)Cl)NC2=O)CCCN4C5=CC=CC=C5NC4=O | InChI=1S/C22H24ClN5O2/c23-15-6-7-20-18(14-15)25-22(30)28(20)16-8-12-26(13-9-16)10-3-11-27-19-5-2-1-4-17(19)24-21(27)29/h1-2,4-7,14,16H,3,8-13H2,(H,24,29)(H,25,30) | 3151 | 57808-66-9 |
| **Dothiepin (dosulepine)** | 296,1464709 | CN(C)CCC=C1C2=CC=CC=C2CSC3=CC=CC=C31 | InChI=1S/C19H21NS/c1-20(2)13-7-11-17-16-9-4-3-8-15(16)14-21-19-12-6-5-10-18(17)19/h3-6,8-12H,7,13-14H2,1-2H3/b17-11+ | 5284550 | 113-53-1 |
| **duloxetine** | 298,1257354 | CNCCC(C1=CC=CS1)OC2=CC=CC3=CC=CC=C32 | InChI=1S/C18H19NOS/c1-19-12-11-17(18-10-5-13-21-18)20-16-9-4-7-14-6-2-3-8-15(14)16/h2-10,13,17,19H,11-12H2,1H3/t17-/m0/s1 | 60835 | 116539-59-4 |
| **Ecgonine methyl ester** | 200,1278434 | CN1C2CCC1C(C(C2)O)C(=O)OC | InChI=1S/C10H17NO3/c1-11-6-3-4-7(11)9(8(12)5-6)10(13)14-2/h6-9,12H,3-5H2,1-2H3/t6-,7+,8-,9+/m0/s1 | 104904 | [7143*09*1](https://commonchemistry.cas.org/detail?cas_rn=7143-09-1&search=7143091) |
| **Enalaprilat** | 349,1755219 | CC(C(=O)N1CCCC1C(=O)O)NC(CCC2=CC=CC=C2)C(=O)O | InChI=1S/C18H24N2O5/c1-12(16(21)20-11-5-8-15(20)18(24)25)19-14(17(22)23)10-9-13-6-3-2-4-7-13/h2-4,6-7,12,14-15,19H,5,8-11H2,1H3,(H,22,23)(H,24,25)/t12-,14-,15-/m0/s1 | 5462501 | 76420-72-9 |
| **Ethylmorphine** | 314,1747936 | CCOC1=C2C3=C(CC4C5C3(CCN4C)C(O2)C(C=C5)O)C=C1 | InChI=1S/C19H23NO3/c1-3-22-15-7-4-11-10-13-12-5-6-14(21)18-19(12,8-9-20(13)2)16(11)17(15)23-18/h4-7,12-14,18,21H,3,8-10H2,1-2H3/t12-,13+,14-,18-,19-/m0/s1 | 5359271 | 76-58-4 |
| **fentanyl** | 337,2271635 | CCC(=O)N(C1CCN(CC1)CCC2=CC=CC=C2)C3=CC=CC=C3 | InChI=1S/C22H28N2O/c1-2-22(25)24(20-11-7-4-8-12-20)21-14-17-23(18-15-21)16-13-19-9-5-3-6-10-19/h3-12,21H,2,13-18H2,1H3 | 3345 | 437-38-7 |
| **flecainide** | 415,1448115 | C1CCNC(C1)CNC(=O)C2=C(C=CC(=C2)OCC(F)(F)F)OCC(F)(F)F | InChI=1S/C17H20F6N2O3/c18-16(19,20)9-27-12-4-5-14(28-10-17(21,22)23)13(7-12)15(26)25-8-11-3-1-2-6-24-11/h4-5,7,11,24H,1-3,6,8-10H2,(H,25,26) | 3356 | 54143-55-4 |
| **fluconazole** | 307,1110654 | C1=CC(=C(C=C1F)F)C(CN2C=NC=N2)(CN3C=NC=N3)O | InChI=1S/C13H12F2N6O/c14-10-1-2-11(12(15)3-10)13(22,4-20-8-16-6-18-20)5-21-9-17-7-19-21/h1-3,6-9,22H,4-5H2 | 3365 | 86386-73-4 |
| **flunitrazepam** | 314,0932694 | CN1C(=O)CN=C(C2=C1C=CC(=C2)[N+](=O)[O-])C3=CC=CC=C3F | InChI=1S/C16H12FN3O3/c1-19-14-7-6-10(20(22)23)8-12(14)16(18-9-15(19)21)11-4-2-3-5-13(11)17/h2-8H,9H2,1H3 | 3380 | 1622-62-4 |
| **fluoxetine** | 310,1410487 | CNCCC(C1=CC=CC=C1)OC2=CC=C(C=C2)C(F)(F)F | InChI=1S/C17H18F3NO/c1-21-12-11-16(13-5-3-2-4-6-13)22-15-9-7-14(8-10-15)17(18,19)20/h2-10,16,21H,11-12H2,1H3 | 3386 | 54910-89-3 |
| **ganciclovir** | 256,1037539 | C1=NC2=C(N1COC(CO)CO)N=C(NC2=O)N | InChI=1S/C9H13N5O4/c10-9-12-7-6(8(17)13-9)11-3-14(7)4-18-5(1-15)2-16/h3,5,15-16H,1-2,4H2,(H3,10,12,13,17) | 135398740 | 82410-32-0 |
| **gliclazide** | 324,1373627 | CC1=CC=C(C=C1)S(=O)(=O)NC(=O)NN2CC3CCCC3C2 | InChI=1S/C15H21N3O3S/c1-11-5-7-14(8-6-11)22(20,21)17-15(19)16-18-9-12-3-2-4-13(12)10-18/h5-8,12-13H,2-4,9-10H2,1H3,(H2,16,17,19) | 3475 | 21187-98-4 |
| **haloperidol** | 376,1471348 | C1CN(CCC1(C2=CC=C(C=C2)Cl)O)CCCC(=O)C3=CC=C(C=C3)F | InChI=1S/C21H23ClFNO2/c22-18-7-5-17(6-8-18)21(26)11-14-24(15-12-21)13-1-2-20(25)16-3-9-19(23)10-4-16/h3-10,26H,1-2,11-15H2 | 3559 | 52-86-8 |
| **hydromorphone** | 286,1434935 | CN1CCC23C4C1CC5=C2C(=C(C=C5)O)OC3C(=O)CC4 | InChI=1S/C17H19NO3/c1-18-7-6-17-10-3-5-13(20)16(17)21-15-12(19)4-2-9(14(15)17)8-11(10)18/h2,4,10-11,16,19H,3,5-8H2,1H3/t10-,11+,16-,17-/m0/s1 | 5284570 | 466-99-9 |
| **hydroxychloroquine** | 336,1834402 | CCN(CCCC(C)NC1=C2C=CC(=CC2=NC=C1)Cl)CCO | InChI=1S/C18H26ClN3O/c1-3-22(11-12-23)10-4-5-14(2)21-17-8-9-20-18-13-15(19)6-7-16(17)18/h6-9,13-14,23H,3-5,10-12H2,1-2H3,(H,20,21) | 3652 | 118-42-3 |
| **hydroxyurea** | 77,03427738 | C(=O)(N)NO | InChI=1S/CH4N2O2/c2-1(4)3-5/h5H,(H3,2,3,4) | 3657 | 127-07-1 |
| **imipramine** | 281,2009488 | CN(C)CCCN1C2=CC=CC=C2CCC3=CC=CC=C31 | InChI=1S/C19H24N2/c1-20(2)14-7-15-21-18-10-5-3-8-16(18)12-13-17-9-4-6-11-19(17)21/h3-6,8-11H,7,12-15H2,1-2H3 | 3696 | 50-49-7 |
| **isoniazid** | 138,0659119 | C1=CN=CC=C1C(=O)NN | InChI=1S/C6H7N3O/c7-9-6(10)5-1-3-8-4-2-5/h1-4H,7H2,(H,9,10) | 3767 | 54-85-3 |
| **Ivabradine** | 469,2694223 | CN(CCCN1CCC2=CC(=C(C=C2CC1=O)OC)OC)CC3CC4=CC(=C(C=C34)OC)OC | InChI=1S/C27H36N2O5/c1-28(17-21-11-20-14-25(33-4)26(34-5)16-22(20)21)8-6-9-29-10-7-18-12-23(31-2)24(32-3)13-19(18)15-27(29)30/h12-14,16,21H,6-11,15,17H2,1-5H3/t21-/m1/s1 | 132999 | 155974-00-8 |
| **labetalol** | 329,1856926 | CC(CCC1=CC=CC=C1)NCC(C2=CC(=C(C=C2)O)C(=O)N)O | InChI=1S/C19H24N2O3/c1-13(7-8-14-5-3-2-4-6-14)21-12-18(23)15-9-10-17(22)16(11-15)19(20)24/h2-6,9-11,13,18,21-23H,7-8,12H2,1H3,(H2,20,24) | 3869 | 36894-69-6 |
| **lamivudine** | 230,0591124 | C1C(OC(S1)CO)N2C=CC(=NC2=O)N | InChI=1S/C8H11N3O3S/c9-5-1-2-11(8(13)10-5)6-4-15-7(3-12)14-6/h1-2,6-7,12H,3-4H2,(H2,9,10,13)/t6-,7+/m0/s1 | 60825 | 134678-17-4 |
| **lansoprazole** | 370,0828824 | CC1=C(C=CN=C1CS(=O)C2=NC3=CC=CC=C3N2)OCC(F)(F)F | InChI=1S/C16H14F3N3O2S/c1-10-13(20-7-6-14(10)24-9-16(17,18)19)8-25(23)15-21-11-4-2-3-5-12(11)22-15/h2-7H,8-9H2,1H3,(H,21,22) | 3883 | 103577-45-3 |
| **levamisole** | 205,0791196 | C1CSC2=NC(CN21)C3=CC=CC=C3 | InChI=1S/C11H12N2S/c1-2-4-9(5-3-1)10-8-13-6-7-14-11(13)12-10/h1-5,10H,6-8H2/t10-/m1/s1 | 26879 | 14769-73-4 |
| **Levomepromazine** | 329,1679346 | CC(CN1C2=CC=CC=C2SC3=C1C=C(C=C3)OC)CN(C)C | InChI=1S/C19H24N2OS/c1-14(12-20(2)3)13-21-16-7-5-6-8-18(16)23-19-10-9-15(22-4)11-17(19)21/h5-11,14H,12-13H2,1-4H3/t14-/m1/s1 | 72287 | 60-99-1 |
| **lidocaine** | 235,1802133 | CCN(CC)CC(=O)NC1=C(C=CC=C1C)C | InChI=1S/C14H22N2O/c1-5-16(6-2)10-13(17)15-14-11(3)8-7-9-12(14)4/h7-9H,5-6,10H2,1-4H3,(H,15,17) | 3676 | 137-58-6 |
| **loperamide** | 477,230056 | CN(C)C(=O)C(CCN1CCC(CC1)(C2=CC=C(C=C2)Cl)O)(C3=CC=CC=C3)C4=CC=CC=C4 | InChI=1S/C29H33ClN2O2/c1-31(2)27(33)29(24-9-5-3-6-10-24,25-11-7-4-8-12-25)19-22-32-20-17-28(34,18-21-32)23-13-15-26(30)16-14-23/h3-16,34H,17-22H2,1-2H3 | 3955 | 53179-11-6 |
| **lorazepam** | 321,018933 | C1=CC=C(C(=C1)C2=NC(C(=O)NC3=C2C=C(C=C3)Cl)O)Cl | InChI=1S/C15H10Cl2N2O2/c16-8-5-6-12-10(7-8)13(19-15(21)14(20)18-12)9-3-1-2-4-11(9)17/h1-7,15,21H,(H,18,20) | 3958 | 846-49-1 |
| **Lormetazepam** | 335,034583 | CN1C2=C(C=C(C=C2)Cl)C(=NC(C1=O)O)C3=CC=CC=C3Cl | InChI=1S/C16H12Cl2N2O2/c1-20-13-7-6-9(17)8-11(13)14(19-15(21)16(20)22)10-4-2-3-5-12(10)18/h2-8,15,21H,1H3 | 13314 | 848-75-9 |
| **loxapine** | 328,1208399 | CN1CCN(CC1)C2=NC3=CC=CC=C3OC4=C2C=C(C=C4)Cl | InChI=1S/C18H18ClN3O/c1-21-8-10-22(11-9-21)18-14-12-13(19)6-7-16(14)23-17-5-3-2-4-15(17)20-18/h2-7,12H,8-11H2,1H3 | 3964 | 1977*10*2 |
| **Lysergide** | 324,2067624 | CCN(CC)C(=O)C1CN(C2CC3=CNC4=CC=CC(=C34)C2=C1)C | InChI=1S/C20H25N3O/c1-4-23(5-2)20(24)14-9-16-15-7-6-8-17-19(15)13(11-21-17)10-18(16)22(3)12-14/h6-9,11,14,18,21H,4-5,10,12H2,1-3H3/t14-,18-/m1/s1 | 5761 | 50-37-3 |
| **maprotiline** | 278,1900498 | CNCCCC12CCC(C3=CC=CC=C31)C4=CC=CC=C24 | InChI=1S/C20H23N/c1-21-14-6-12-20-13-11-15(16-7-2-4-9-18(16)20)17-8-3-5-10-19(17)20/h2-5,7-10,15,21H,6,11-14H2,1H3 | 4011 | 10262-69-8 |
| **MDEA** | 208,1329288 | CCNC(C)CC1=CC2=C(C=C1)OCO2 | InChI=1S/C12H17NO2/c1-3-13-9(2)6-10-4-5-11-12(7-10)15-8-14-11/h4-5,7,9,13H,3,6,8H2,1-2H3 | 105039 | 82801-81-8 |
| **MDMA** | 194,1172787 | CC(CC1=CC2=C(C=C1)OCO2)NC | InChI=1S/C11H15NO2/c1-8(12-2)5-9-3-4-10-11(6-9)14-7-13-10/h3-4,6,8,12H,5,7H2,1-2H3 | 1615 | 42542-10-9 |
| **medazepam** | 271,0993762 | CN1CCN=C(C2=C1C=CC(=C2)Cl)C3=CC=CC=C3 | InChI=1S/C16H15ClN2/c1-19-10-9-18-16(12-5-3-2-4-6-12)14-11-13(17)7-8-15(14)19/h2-8,11H,9-10H2,1H3 | 4041 | 364854 |
| **mepivacaine** | 247,1802133 | CC1=C(C(=CC=C1)C)NC(=O)C2CCCCN2C | InChI=1S/C15H22N2O/c1-11-7-6-8-12(2)14(11)16-15(18)13-9-4-5-10-17(13)3/h6-8,13H,4-5,9-10H2,1-3H3,(H,16,18) | 4062 | 96-88-8 |
| **meprobamate** | 219,1336571 | CCCC(C)(COC(=O)N)COC(=O)N | InChI=1S/C9H18N2O4/c1-3-4-9(2,5-14-7(10)12)6-15-8(11)13/h3-6H2,1-2H3,(H2,10,12)(H2,11,13) | 4064 | 57-53-4 |
| **meropenem** | 384,1584921 | CC1C2C(C(=O)N2C(=C1SC3CC(NC3)C(=O)N(C)C)C(=O)O)C(C)O | InChI=1S/C17H25N3O5S/c1-7-12-11(8(2)21)16(23)20(12)13(17(24)25)14(7)26-9-5-10(18-6-9)15(22)19(3)4/h7-12,18,21H,5-6H2,1-4H3,(H,24,25)/t7-,8-,9+,10+,11-,12-/m1/s1 | 441130 | 96036-03-2 |
| **metamphetamine** | 150,1274495 | CC(CC1=CC=CC=C1)NC | InChI=1S/C10H15N/c1-9(11-2)8-10-6-4-3-5-7-10/h3-7,9,11H,8H2,1-2H3/t9-/m0/s1 | 10836 | 537-46-2 |
| **methadone** | 310,2162645 | CCC(=O)C(CC(C)N(C)C)(C1=CC=CC=C1)C2=CC=CC=C2 | InChI=1S/C21H27NO/c1-5-20(23)21(16-17(2)22(3)4,18-12-8-6-9-13-18)19-14-10-7-11-15-19/h6-15,17H,5,16H2,1-4H3 | 4095 | 76-99-3 |
| **Methoxetamine** | 248,1642289 | CCNC1(CCCCC1=O)C2=CC(=CC=C2)OC | InChI=1S/C15H21NO2/c1-3-16-15(10-5-4-9-14(15)17)12-7-6-8-13(11-12)18-2/h6-8,11,16H,3-5,9-10H2,1-2H3 | 52911279 | 1239943-76-0 |
| **Methylenedioxypyrovalerone (MDPV)** | 276,1591435 | CCCC(C(=O)C1=CC2=C(C=C1)OCO2)N3CCCC3 | InChI=1S/C16H21NO3/c1-2-5-13(17-8-3-4-9-17)16(18)12-6-7-14-15(10-12)20-11-19-14/h6-7,10,13H,2-5,8-9,11H2,1H3 | 20111961 | 687603-66-3 |
| **methylone** | 208,0965433 | CC(C(=O)C1=CC2=C(C=C1)OCO2)NC | InChI=1S/C11H13NO3/c1-7(12-2)11(13)8-3-4-9-10(5-8)15-6-14-9/h3-5,7,12H,6H2,1-2H3 | 45789647 | 186028-79-5 |
| **metoclopramide** | 300,1470547 | CCN(CC)CCNC(=O)C1=CC(=C(C=C1OC)N)Cl | InChI=1S/C14H22ClN3O2/c1-4-18(5-2)7-6-17-14(19)10-8-11(15)12(16)9-13(10)20-3/h8-9H,4-7,16H2,1-3H3,(H,17,19) | 4168 | 364-62-5 |
| **metoprolol** | 268,1904437 | CC(C)NCC(COC1=CC=C(C=C1)CCOC)O | InChI=1S/C15H25NO3/c1-12(2)16-10-14(17)11-19-15-6-4-13(5-7-15)8-9-18-3/h4-7,12,14,16-17H,8-11H2,1-3H3 | 4171 | 51384-51-1 |
| **metronidazole** | 172,0713912 | CC1=NC=C(N1CCO)[N+](=O)[O-] | InChI=1S/C6H9N3O3/c1-5-7-4-6(9(11)12)8(5)2-3-10/h4,10H,2-3H2,1H3 | 4173 | 443-48-1 |
| **mianserin** | 265,1696487 | CN1CCN2C(C1)C3=CC=CC=C3CC4=CC=CC=C42 | InChI=1S/C18H20N2/c1-19-10-11-20-17-9-5-3-7-15(17)12-14-6-2-4-8-16(14)18(20)13-19/h2-9,18H,10-13H2,1H3 | 4184 | 24219-97-4 |
| **midazolam** | 326,0852033 | CC1=NC=C2N1C3=C(C=C(C=C3)Cl)C(=NC2)C4=CC=CC=C4F | InChI=1S/C18H13ClFN3/c1-11-21-9-13-10-22-18(14-4-2-3-5-16(14)20)15-8-12(19)6-7-17(15)23(11)13/h2-9H,10H2,1H3 | 4192 | 59467-70-8 |
| **mirtazapine** | 266,1648976 | CN1CCN2C(C1)C3=CC=CC=C3CC4=C2N=CC=C4 | InChI=1S/C17H19N3/c1-19-9-10-20-16(12-19)15-7-3-2-5-13(15)11-14-6-4-8-18-17(14)20/h2-8,16H,9-12H2,1H3 | 4205 | 85650-52-8 |
| **morphine** | 286,1434935 | CN1CCC23C4C1CC5=C2C(=C(C=C5)O)OC3C(C=C4)O | InChI=1S/C17H19NO3/c1-18-7-6-17-10-3-5-13(20)16(17)21-15-12(19)4-2-9(14(15)17)8-11(10)18/h2-5,10-11,13,16,19-20H,6-8H2,1H3/t10-,11+,13-,16-,17-/m0/s1 | 5288826 | 57-27-2 |
| **N-[2-(2-Carbamoylmethoxy-Ethoxy)-Ethyl]-2-[2-(4-Chloro-Phenylsulfanyl)-Acetylamino]-3-(4-Guanidino-Phenyl)-Propionamide (2-CE)** | 551,183517 | C1=CC(=CC=C1CC(C(=O)NCCOCCOCC(=O)N)NC(=O)CSC2=CC=C(C=C2)Cl)N=C(N)N | InChI=1S/C24H31ClN6O5S/c25-17-3-7-19(8-4-17)37-15-22(33)31-20(13-16-1-5-18(6-2-16)30-24(27)28)23(34)29-9-10-35-11-12-36-14-21(26)32/h1-8,20H,9-15H2,(H2,26,32)(H,29,34)(H,31,33)(H4,27,28,30)/t20-/m0/s1 | 11957381 | 71539-34-9 |
| **Nebivolol** | 406,1821646 | C1CC2=C(C=CC(=C2)F)OC1C(CNCC(C3CCC4=C(O3)C=CC(=C4)F)O)O | InChI=1S/C22H25F2NO4/c23-15-3-7-19-13(9-15)1-5-21(28-19)17(26)11-25-12-18(27)22-6-2-14-10-16(24)4-8-20(14)29-22/h3-4,7-10,17-18,21-22,25-27H,1-2,5-6,11-12H2 | 71301 | 99200-09-6 |
| **nevirapine** | 267,1237611 | CC1=C2C(=NC=C1)N(C3=C(C=CC=N3)C(=O)N2)C4CC4 | InChI=1S/C15H14N4O/c1-9-6-8-17-14-12(9)18-15(20)11-3-2-7-16-13(11)19(14)10-4-5-10/h2-3,6-8,10H,4-5H2,1H3,(H,18,20) | 4463 | 129618-40-2 |
| **nicardipine** | 480,2126357 | CC1=C(C(C(=C(N1)C)C(=O)OCCN(C)CC2=CC=CC=C2)C3=CC(=CC=C3)[N+](=O)[O-])C(=O)OC | InChI=1S/C26H29N3O6/c1-17-22(25(30)34-4)24(20-11-8-12-21(15-20)29(32)33)23(18(2)27-17)26(31)35-14-13-28(3)16-19-9-6-5-7-10-19/h5-12,15,24,27H,13-14,16H2,1-4H3 | 4474 | 55985-32-5 |
| **nifedipine** | 347,1234863 | CC1=C(C(C(=C(N1)C)C(=O)OC)C2=CC=CC=C2[N+](=O)[O-])C(=O)OC | InChI=1S/C17H18N2O6/c1-9-13(16(20)24-3)15(14(10(2)18-9)17(21)25-4)11-7-5-6-8-12(11)19(22)23/h5-8,15,18H,1-4H3 | 4485 | 21829-25-4 |
| **norbuprenorphine** | 414,2636086 | CC(C)(C)C(C)(C1CC23CCC1(C4C25CCNC3CC6=C5C(=C(C=C6)O)O4)OC)O | InChI=1S/C25H35NO4/c1-21(2,3)22(4,28)16-13-23-8-9-25(16,29-5)20-24(23)10-11-26-17(23)12-14-6-7-15(27)19(30-20)18(14)24/h6-7,16-17,20,26-28H,8-13H2,1-5H3/t16-,17-,20-,22+,23-,24+,25-/m1/s1 | 114976 | 78715-23-8 |
| **nordiazepam** | 271,0629907 | C1C(=O)NC2=C(C=C(C=C2)Cl)C(=N1)C3=CC=CC=C3 | InChI=1S/C15H11ClN2O/c16-11-6-7-13-12(8-11)15(17-9-14(19)18-13)10-4-2-1-3-5-10/h1-8H,9H2,(H,18,19) | 2997 | 1088-11-5 |
| **normorphine** | 272,1278434 | C1CNC2CC3=C4C15C2C=CC(C5OC4=C(C=C3)O)O | InChI=1S/C16H17NO3/c18-11-3-1-8-7-10-9-2-4-12(19)15-16(9,5-6-17-10)13(8)14(11)20-15/h1-4,9-10,12,15,17-19H,5-7H2/t9-,10+,12-,15-,16-/m0/s1 | 5462508 | 466-97-7 |
| **nortriptyline** | 264,1743997 | CNCCC=C1C2=CC=CC=C2CCC3=CC=CC=C31 | InChI=1S/C19H21N/c1-20-14-6-11-19-17-9-4-2-7-15(17)12-13-16-8-3-5-10-18(16)19/h2-5,7-11,20H,6,12-14H2,1H3 | 4543 | 72-69-5 |
| **norverapamil** | 441,2745077 | CC(C)C(CCCNCCC1=CC(=C(C=C1)OC)OC)(C#N)C2=CC(=C(C=C2)OC)OC | InChI=1S/C26H36N2O4/c1-19(2)26(18-27,21-9-11-23(30-4)25(17-21)32-6)13-7-14-28-15-12-20-8-10-22(29-3)24(16-20)31-5/h8-11,16-17,19,28H,7,12-15H2,1-6H3 | 104972 | 67018-85-3 |
| **O-Desmethyltramadol** | 250,179879 | CN(C)CC1CCCCC1(C2=CC(=CC=C2)O)O | InChI=1S/C15H23NO2/c1-16(2)11-13-6-3-4-9-15(13,18)12-7-5-8-14(17)10-12/h5,7-8,10,13,17-18H,3-4,6,9,11H2,1-2H3/t13-,15+/m1/s1 | 9838803 | 80456-81-1 |
| **ofloxacin** | 362,1507843 | CC1COC2=C3N1C=C(C(=O)C3=CC(=C2N4CCN(CC4)C)F)C(=O)O | InChI=1S/C18H20FN3O4/c1-10-9-26-17-14-11(16(23)12(18(24)25)8-22(10)14)7-13(19)15(17)21-5-3-20(2)4-6-21/h7-8,10H,3-6,9H2,1-2H3,(H,24,25) | 4583 | 82419-36-1 |
| **omeprazole** | 346,1217127 | CC1=CN=C(C(=C1OC)C)CS(=O)C2=NC3=C(N2)C=C(C=C3)OC | InChI=1S/C17H19N3O3S/c1-10-8-18-15(11(2)16(10)23-4)9-24(21)17-19-13-6-5-12(22-3)7-14(13)20-17/h5-8H,9H2,1-4H3,(H,19,20) | 4594 | 73590-58-6 |
| **oxazepam** | 287,0579053 | C1=CC=C(C=C1)C2=NC(C(=O)NC3=C2C=C(C=C3)Cl)O | InChI=1S/C15H11ClN2O2/c16-10-6-7-12-11(8-10)13(9-4-2-1-3-5-9)18-15(20)14(19)17-12/h1-8,15,20H,(H,17,19) | 4616 | 604-75-1 |
| **oxycodone** | 316,1540582 | CN1CCC23C4C(=O)CCC2(C1CC5=C3C(=C(C=C5)OC)O4)O | InChI=1S/C18H21NO4/c1-19-8-7-17-14-10-3-4-12(22-2)15(14)23-16(17)11(20)5-6-18(17,21)13(19)9-10/h3-4,13,16,21H,5-9H2,1-2H3/t13-,16+,17+,18-/m1/s1 | 5284603 | 76-42-6 |
| **paroxetine** | 330,1497217 | C1CNCC(C1C2=CC=C(C=C2)F)COC3=CC4=C(C=C3)OCO4 | InChI=1S/C19H20FNO3/c20-15-3-1-13(2-4-15)17-7-8-21-10-14(17)11-22-16-5-6-18-19(9-16)24-12-23-18/h1-6,9,14,17,21H,7-8,10-12H2/t14-,17-/m0/s1 | 43815 | 61869-08-7 |
| **pholcodineE** | 399,2275575 | CN1CCC23C4C1CC5=C2C(=C(C=C5)OCCN6CCOCC6)OC3C(C=C4)O | InChI=1S/C23H30N2O4/c1-24-7-6-23-16-3-4-18(26)22(23)29-21-19(5-2-15(20(21)23)14-17(16)24)28-13-10-25-8-11-27-12-9-25/h2-5,16-18,22,26H,6-14H2,1H3/t16-,17+,18-,22-,23-/m0/s1 | 5311356 | 509-67-1 |
| **piperacillin** | 518,1701194 | CCN1CCN(C(=O)C1=O)C(=O)NC(C2=CC=CC=C2)C(=O)NC3C4N(C3=O)C(C(S4)(C)C)C(=O)O | InChI=1S/C23H27N5O7S/c1-4-26-10-11-27(19(32)18(26)31)22(35)25-13(12-8-6-5-7-9-12)16(29)24-14-17(30)28-15(21(33)34)23(2,3)36-20(14)28/h5-9,13-15,20H,4,10-11H2,1-3H3,(H,24,29)(H,25,35)(H,33,34)/t13-,14-,15+,20-/m1/s1 | 43672 | 61477-96-1 |
| **prednisone** | 359,1850239 | CC12CC(=O)C3C(C1CCC2(C(=O)CO)O)CCC4=CC(=O)C=CC34C | InChI=1S/C21H26O5/c1-19-7-5-13(23)9-12(19)3-4-14-15-6-8-21(26,17(25)11-22)20(15,2)10-16(24)18(14)19/h5,7,9,14-15,18,22,26H,3-4,6,8,10-11H2,1-2H3/t14-,15-,18+,19-,20-,21-/m0/s1 | 5865 | 53-03-2 |
| **pristinamycin** | 1349,608591 | CCC1C(=O)N2CCCC2C(=O)N(C(C(=O)N3CCC(=O)CC3C(=O)NC(C(=O)OC(C(C(=O)N1)NC(=O)C4=C(C=CC=N4)O)C)C5=CC=CC=C5)CC6=CC=CC=C6)C.CC1C=CC(=O)NCC=CC(=CC(CC(=O)CC2=NC(=CO2)C(=O)N3CCC=C3C(=O)OC1C(C)C)O)C | InChI=1S/C43H49N7O10.C28H35N3O7/c1-4-29-40(56)49-21-12-17-30(49)41(57)48(3)32(23-26-13-7-5-8-14-26)42(58)50-22-19-28(51)24-31(50)37(53)47-35(27-15-9-6-10-16-27)43(59)60-25(2)34(38(54)45-29)46-39(55)36-33(52)18-11-20-44-36;1-17(2)26-19(4)9-10-24(34)29-11-5-7-18(3)13-20(32)14-21(33)15-25-30-22(16-37-25)27(35)31-12-6-8-23(31)28(36)38-26/h5-11,13-16,18,20,25,29-32,34-35,52H,4,12,17,19,21-24H2,1-3H3,(H,45,54)(H,46,55)(H,47,53);5,7-10,13,16-17,19-20,26,32H,6,11-12,14-15H2,1-4H3,(H,29,34)/b;7-5-,10-9-,18-13- | 11979535 | 11006-76-1 |
| **propranolol** | 260,1642289 | CC(C)NCC(COC1=CC=CC2=CC=CC=C21)O | InChI=1S/C16H21NO2/c1-12(2)17-10-14(18)11-19-16-9-5-7-13-6-3-4-8-15(13)16/h3-9,12,14,17-18H,10-11H2,1-2H3 | 4946 | 525-66-6 |
| **pyrazinamide** | 124,0502618 | C1=CN=C(C=N1)C(=O)N | InChI=1S/C5H5N3O/c6-5(9)4-3-7-1-2-8-4/h1-3H,(H2,6,9) | 1046 | 98-96-4 |
| **pyrimethamine** | 249,0898741 | CCC1=C(C(=NC(=N1)N)N)C2=CC=C(C=C2)Cl | InChI=1S/C12H13ClN4/c1-2-9-10(11(14)17-12(15)16-9)7-3-5-8(13)6-4-7/h3-6H,2H2,1H3,(H4,14,15,16,17) | 4993 | 58-14-0 |
| **quetiapine** | 384,1737482 | C1CN(CCN1CCOCCO)C2=NC3=CC=CC=C3SC4=CC=CC=C42 | InChI=1S/C21H25N3O2S/c25-14-16-26-15-13-23-9-11-24(12-10-23)21-17-5-1-3-7-19(17)27-20-8-4-2-6-18(20)22-21/h1-8,25H,9-16H2 | 5002 | 111974-69-7 |
| **quinine** | 325,190778 | COC1=CC2=C(C=CN=C2C=C1)C(C3CC4CCN3CC4C=C)O | InChI=1S/C20H24N2O2/c1-3-13-12-22-9-7-14(13)10-19(22)20(23)16-6-8-21-18-5-4-15(24-2)11-17(16)18/h3-6,8,11,13-14,19-20,23H,1,7,9-10,12H2,2H3/t13-,14-,19-,20+/m0/s1 | 3034034 | 130-95-0 |
| **rivaroxaban** | 436,0725696 | C1COCC(=O)N1C2=CC=C(C=C2)N3CC(OC3=O)CNC(=O)C4=CC=C(S4)Cl | InChI=1S/C19H18ClN3O5S/c20-16-6-5-15(29-16)18(25)21-9-14-10-23(19(26)28-14)13-3-1-12(2-4-13)22-7-8-27-11-17(22)24/h1-6,14H,7-11H2,(H,21,25)/t14-/m0/s1 | 9875401 | 366789-02-8 |
| **ropivacaine** | 275,2115135 | CCCN1CCCCC1C(=O)NC2=C(C=CC=C2C)C | InChI=1S/C17H26N2O/c1-4-11-19-12-6-5-10-15(19)17(20)18-16-13(2)8-7-9-14(16)3/h7-9,15H,4-6,10-12H2,1-3H3,(H,18,20)/t15-/m0/s1 | 175805 | 84057-95-4 |
| **sertraline** | 306,0808049 | CNC1CCC(C2=CC=CC=C12)C3=CC(=C(C=C3)Cl)Cl | InChI=1S/C17H17Cl2N/c1-20-17-9-7-12(13-4-2-3-5-14(13)17)11-6-8-15(18)16(19)10-11/h2-6,8,10,12,17,20H,7,9H2,1H3/t12-,17-/m0/s1 | 68617 | 79617-96-2 |
| **sotalol** | 273,1264637 | CC(C)NCC(C1=CC=C(C=C1)NS(=O)(=O)C)O | InChI=1S/C12H20N2O3S/c1-9(2)13-8-12(15)10-4-6-11(7-5-10)14-18(3,16)17/h4-7,9,12-15H,8H2,1-3H3 | 5253 | 3930-20-9 |
| **sufentanil (sulfentanyl)** | 387,2097994 | CCC(=O)N(C1=CC=CC=C1)C2(CCN(CC2)CCC3=CC=CS3)COC | InChI=1S/C22H30N2O2S/c1-3-21(25)24(19-8-5-4-6-9-19)22(18-26-2)12-15-23(16-13-22)14-11-20-10-7-17-27-20/h4-10,17H,3,11-16,18H2,1-2H3 | 41693 | 56030-54-7 |
| **sulfadiazine** | 251,0594468 | C1=CN=C(N=C1)NS(=O)(=O)C2=CC=C(C=C2)N | InChI=1S/C10H10N4O2S/c11-8-2-4-9(5-3-8)17(15,16)14-10-12-6-1-7-13-10/h1-7H,11H2,(H,12,13,14) | 5215 | 68-35-9 |
| **sulfamethoxazole** | 254,0591124 | CC1=CC(=NO1)NS(=O)(=O)C2=CC=C(C=C2)N | InChI=1S/C10H11N3O3S/c1-7-6-10(12-16-7)13-17(14,15)9-4-2-8(11)3-5-9/h2-6H,11H2,1H3,(H,12,13) | 5329 | 723-46-6 |
| **temazepam** | 301,0735554 | CN1C2=C(C=C(C=C2)Cl)C(=NC(C1=O)O)C3=CC=CC=C3 | InChI=1S/C16H13ClN2O2/c1-19-13-8-7-11(17)9-12(13)14(18-15(20)16(19)21)10-5-3-2-4-6-10/h2-9,15,20H,1H3 | 5391 | 846-50-4 |
| **tocainide** | 193,1332631 | CC1=C(C(=CC=C1)C)NC(=O)C(C)N | InChI=1S/C11H16N2O/c1-7-5-4-6-8(2)10(7)13-11(14)9(3)12/h4-6,9H,12H2,1-3H3,(H,13,14) | 38945 | 41708-72-9 |
| **tramadol** | 264,1955291 | CN(C)CC1CCCCC1(C2=CC(=CC=C2)OC)O | InChI=1S/C16H25NO2/c1-17(2)12-14-7-4-5-10-16(14,18)13-8-6-9-15(11-13)19-3/h6,8-9,11,14,18H,4-5,7,10,12H2,1-3H3/t14-,16+/m1/s1 | 33741 | 27203-92-5 |
| **triazolam** | 343,0509018 | CC1=NN=C2N1C3=C(C=C(C=C3)Cl)C(=NC2)C4=CC=CC=C4Cl | InChI=1S/C17H12Cl2N4/c1-10-21-22-16-9-20-17(12-4-2-3-5-14(12)19)13-8-11(18)6-7-15(13)23(10)16/h2-8H,9H2,1H3 | 5556 | 28911-01-5 |
| **trimeprazine (Alimemazine)** | 299,1573699 | CC(CN1C2=CC=CC=C2SC3=CC=CC=C31)CN(C)C | InChI=1S/C18H22N2S/c1-14(12-19(2)3)13-20-15-8-4-6-10-17(15)21-18-11-7-5-9-16(18)20/h4-11,14H,12-13H2,1-3H3 | 5574 | 84-96-8 |
| **trimethoprim** | 291,1448905 | COC1=CC(=CC(=C1OC)OC)CC2=CN=C(N=C2N)N | InChI=1S/C14H18N4O3/c1-19-10-5-8(6-11(20-2)12(10)21-3)4-9-7-17-14(16)18-13(9)15/h5-7H,4H2,1-3H3,(H4,15,16,17,18) | 5578 | 738-70-5 |
| **trimipramine** | 295,2165989 | CC(CN1C2=CC=CC=C2CCC3=CC=CC=C31)CN(C)C | InChI=1S/C20H26N2/c1-16(14-21(2)3)15-22-19-10-6-4-8-17(19)12-13-18-9-5-7-11-20(18)22/h4-11,16H,12-15H2,1-3H3 | 5584 | 739-71-9 |
| **urapidil** | 388,2340398 | CN1C(=CC(=O)N(C1=O)C)NCCCN2CCN(CC2)C3=CC=CC=C3OC | InChI=1S/C20H29N5O3/c1-22-18(15-19(26)23(2)20(22)27)21-9-6-10-24-11-13-25(14-12-24)16-7-4-5-8-17(16)28-3/h4-5,7-8,15,21H,6,9-14H2,1-3H3 | 5639 | 34661-75-1 |
| **venlafaxine** | 278,2111791 | CN(C)CC(C1=CC=C(C=C1)OC)C2(CCCCC2)O | InChI=1S/C17H27NO2/c1-18(2)13-16(17(19)11-5-4-6-12-17)14-7-9-15(20-3)10-8-14/h7-10,16,19H,4-6,11-13H2,1-3H3 | 5656 | 93413-69-5 |
| **verapamil** | 455,2901577 | CC(C)C(CCCN(C)CCC1=CC(=C(C=C1)OC)OC)(C#N)C2=CC(=C(C=C2)OC)OC | InChI=1S/C27H38N2O4/c1-20(2)27(19-28,22-10-12-24(31-5)26(18-22)33-7)14-8-15-29(3)16-13-21-9-11-23(30-4)25(17-21)32-6/h9-12,17-18,20H,8,13-16H2,1-7H3 | 2520 | 52-53-9 |
| **viloxazine** | 238,1434935 | CCOC1=CC=CC=C1OCC2CNCCO2 | InChI=1S/C13H19NO3/c1-2-15-12-5-3-4-6-13(12)17-10-11-9-14-7-8-16-11/h3-6,11,14H,2,7-10H2,1H3 | 5666 | 46817-91-8 |
| **voriconazole** | 350,1220446 | CC(C1=NC=NC=C1F)C(CN2C=NC=N2)(C3=C(C=C(C=C3)F)F)O | InChI=1S/C16H14F3N5O/c1-10(15-14(19)5-20-7-22-15)16(25,6-24-9-21-8-23-24)12-3-2-11(17)4-13(12)18/h2-5,7-10,25H,6H2,1H3/t10-,16+/m0/s1 | 71616 | 137234-62-9 |
| **zidovudine (AZT)** | 268,1037539 | CC1=CN(C(=O)NC1=O)C2CC(C(O2)CO)N=[N+]=[N-] | InChI=1S/C10H13N5O4/c1-5-3-15(10(18)12-9(5)17)8-2-6(13-14-11)7(4-16)19-8/h3,6-8,16H,2,4H2,1H3,(H,12,17,18)/t6-,7+,8+/m0/s1 | 35370 | 30516-87-1 |
| **zolpidem** | 308,1754623 | CC1=CC=C(C=C1)C2=C(N3C=C(C=CC3=N2)C)CC(=O)N(C)C | InChI=1S/C19H21N3O/c1-13-5-8-15(9-6-13)19-16(11-18(23)21(3)4)22-12-14(2)7-10-17(22)20-19/h5-10,12H,11H2,1-4H3 | 5732 | 82626-48-0 |
| **zopiclone** | 389,1120661 | CN1CCN(CC1)C(=O)OC2C3=NC=CN=C3C(=O)N2C4=NC=C(C=C4)Cl | InChI=1S/C17H17ClN6O3/c1-22-6-8-23(9-7-22)17(26)27-16-14-13(19-4-5-20-14)15(25)24(16)12-3-2-11(18)10-21-12/h2-5,10,16H,6-9H2,1H3 | 5735 | 43200-80-2 |
